# Ammonia Capture in Rhodium(II)-Based Metal–Organic Polyhedra via Synergistic Coordinative and H-Bonding Interactions

**DOI:** 10.1021/acsami.2c19206

**Published:** 2023-01-25

**Authors:** Arnau Carné-Sánchez, Jordi Martínez-Esaín, Tanner Rookard, Christopher J. Flood, Jordi Faraudo, Kyriakos C. Stylianou, Daniel Maspoch

**Affiliations:** † Catalan Institute of Nanoscience and Nanotechnology (ICN2), CSIC, and Barcelona Institute of Science and Technology, Campus UAB, 08193 Bellaterra, Barcelona, Spain; ‡ Departament de Química, Facultat de Ciències, Universitat Autònoma de Barcelona, 08193 Bellaterra, Spain; § Materials Discovery Laboratory (MaD Lab), Department of Chemistry, Oregon State University, Corvallis, Oregon 97331-4003, United States; ∥ Institut de Ciència de Materials de Barcelona (ICMAB-CSIC), 08193 Bellaterra, Spain; ⊥ ICREA, Pg. Lluís Companys 23, 08010 Barcelona, Spain

**Keywords:** metal−organic polyhedra (MOPs), cages, ammonia capture, molecular dynamics, regeneration

## Abstract

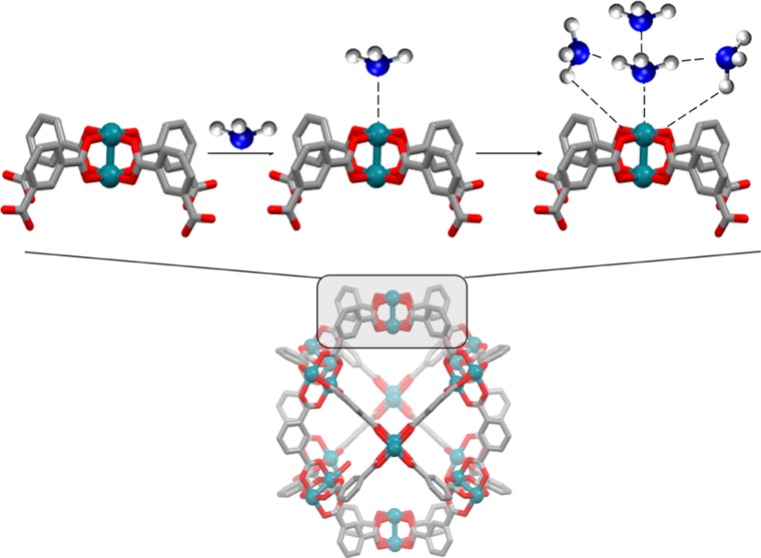

Ammonia (NH_3_) is among the world’s most widely produced bulk chemicals, given its extensive use in diverse sectors such as agriculture; however, it poses environmental and health risks at low concentrations. Therefore, there is a need for developing new technologies and materials to capture and store ammonia safely. Herein, we report for the first time the use of metal–organic polyhedra (MOPs) as ammonia adsorbents. We evaluated three different rhodium-based MOPs: [Rh_2_(bdc)_2_]_12_ (where bdc is 1,3-benzene dicarboxylate); one functionalized with hydroxyl groups at its outer surface [Rh_2_(OH-bdc)_2_]_12_ (where OH-bdc is 5-hydroxy-1,3-benzene dicarboxylate); and one decorated with aliphatic alkoxide chains at its outer surface [Rh_2_(C_12_O-bdc)_2_]_12_ (where C_12_O-bdc is 5-dodecoxybenzene-1,3-benzene dicarboxylate). Ammonia-adsorption experiments revealed that all three Rh-MOPs strongly interact with ammonia, with uptake capacities exceeding 10 mmol/g_MOP_. Furthermore, computational and experimental data showed that the mechanism of the interaction between Rh-MOPs and ammonia proceeds through a first step of coordination of NH_3_ to the axial site of the Rh(II) paddlewheel cluster, which triggers the adsorption of additional NH_3_ molecules through H-bonding interaction. This unique mechanism creates H-bonded clusters of NH_3_ on each Rh(II) axial site, which accounts for the high NH_3_ uptake capacity of Rh-MOPs. Rh-MOPs can be regenerated through their immersion in acidic water, and upon activation, their ammonia uptake can be recovered for at least three cycles. Our findings demonstrate that MOPs can be used as porous hosts to capture corrosive molecules like ammonia, and that their surface functionalization can enhance the ammonia uptake performance.

## 1 Introduction

Ammonia (NH_3_) is industrially produced at an estimated rate of *ca.* 175 million tons per year due to its vital role in the development of fertilizers, which are crucial to securing the food supply chain.^1^ Additionally, ammonia, which is gaseous at room temperature, is increasingly being postulated as an alternative combustion fuel, due to its high energy density (12.7 MJ/L), and as an alternative source for hydrogen, due to its high gravimetric (17.7%) and volumetric (0.105 kg/L) H_2_ energy density.^2^ That said, ammonia is also a pollutant that threatens the environment and human health at a concentration as low as 25 ppm.^3^ Therefore, the development of materials for the safe capture and storage of ammonia is highly desirable to enable the use of NH_3_ as an alternative fuel and to mitigate its harmful environmental and health effects.

Development of stable adsorbents for ammonia has been hindered by its corrosiveness. Initially proposed materials, based on inorganic porous substances such as zeolites, activated carbon, or porous silica, exhibit moderate adsorption capacity (7–9 mmol/g) and lack the structural and compositional versatility for further optimization.^4−8^ Recently, researchers have introduced functional groups with high affinity toward ammonia, including acidic and Lewis-acidic groups, into porous materials such as metal–organic frameworks (MOFs) and covalent-organic frameworks to yield adsorbents with much higher ammonia storage capacity (up to 20 mmol/g) than that of the previously mentioned materials.^9−15^

Metal–organic polyhedra (MOPs) are a sub-class of coordination cages^16,17^ that can store molecules within their cavities, both in the solid state and in solution. Thus, MOPs combine the merits of designed porosity exhibited by reticular materials with the solution processability of monomeric compounds. These features make MOPs an emerging class of adsorbents^18^ that have already demonstrated promise toward the storage and capture of gases such as methane,^19^ carbon dioxide,^20^ carbon monoxide,^21^ nitric oxide,^21^ and sulfur dioxide.^22^ However, given the inherent fragility of most MOPs, which are based on labile coordination bonds [i.e., Cu(II)-carboxylate and Pd(II)-pyridine], they have not been explored as potential porous hosts for ammonia.^23^

**Figure 1:**
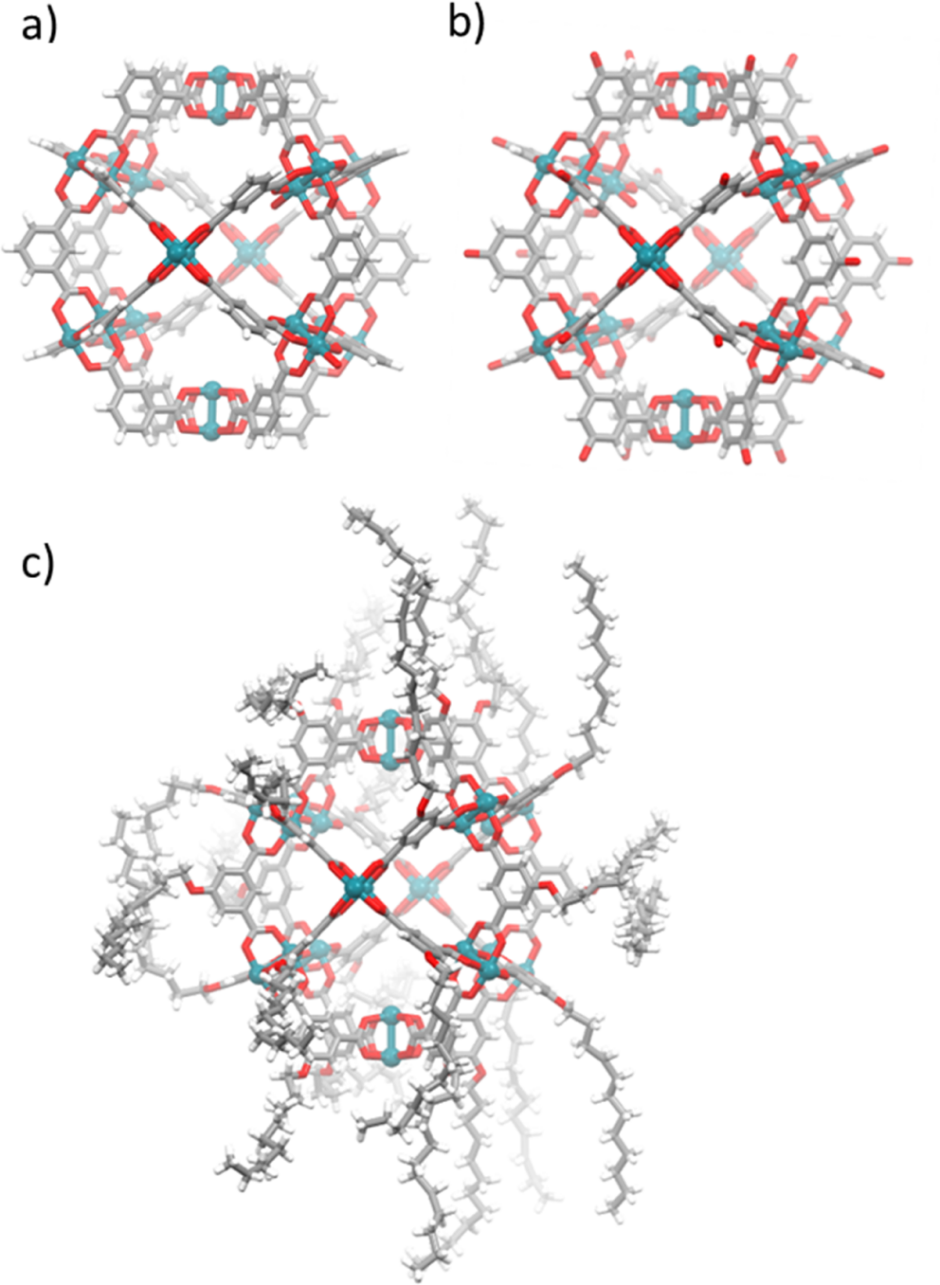
Structures of the three Rh-MOPs included in this study: H-RhMOP (a), OH-RhMOP (b), and C_12_-RhMOP (c). Color code: carbon (gray); hydrogen (white); oxygen (red); and rhodium (blue).

Herein, we introduce the use of MOPs^24^ as adsorbents for ammonia capture. To overcome the limitations related to MOP fragility, we have employed robust cuboctahedral Rh(II)-based MOPs of the general formula [Rh_2_(bdc)_2_]_12_ (where bdc is 1,3-benzene dicarboxylate) as porous media for the uptake of ammonia.^21^ The presence of 12 Rh(II) paddlewheel clusters in the structure of cuboctahedral Rh-MOPs endows them with high chemical and structural stability. Additionally, the desolvation of Rh-MOPs generates open-metal sites at the axial position of the Rh(II) paddlewheel cluster, which can serve as Lewis acid sites to bind NH_3_ molecules.^25^ Thus, Rh-MOPs boast several features that favor the design of adsorbents for ammonia, including microporosity, versatile surface functionalization, and a high density of open-metal sites. We assessed the impact of all these attributes by measuring the ammonia-uptake capacity of the archetypical [Rh_2_(bdc)_2_]_12_ MOP unit (hereafter named H-RhMOP) and two additional surface functionalized MOPs; one functionalized with hydroxyl groups [Rh_2_(OH-bdc)_2_]_12_ (hereafter named OH-RhMOP, where OH-bdc is 5-hydroxy-1,3-benzene dicarboxylate)^26^ and another decorated with aliphatic alkoxide chains [Rh_2_(C_12_O-bdc)_2_]_12_ (hereafter named C_12_-RhMOP where C_12_O-bdc is 5-dodecoxybenzene-1,3-benzene dicarboxylate) (Figures 1 and S1–S4).^27^ Ammonia-adsorption experiments revealed that all three Rh-MOPs strongly interact with ammonia, with uptake capacities exceeding 10 mmol/g. Through computations and experiments, we revealed that the interaction between Rh-MOPs and ammonia proceeds through a first step of coordination of NH_3_ to the axial site of the Rh(II) paddlewheel cluster, which triggers the adsorption of additional NH_3_ molecules through H-bonding interactions. This unique mechanism leads to the generation of H-bonded clusters of NH_3_ on each Rh(II) axial site, which accounts for the high NH_3_ uptake capacity of Rh-MOPs.

## 2 Results and Discussion

### 2.1 Ammonia Adsorption in H-RhMOP: Uptake and Mechanism

**Figure 2:**
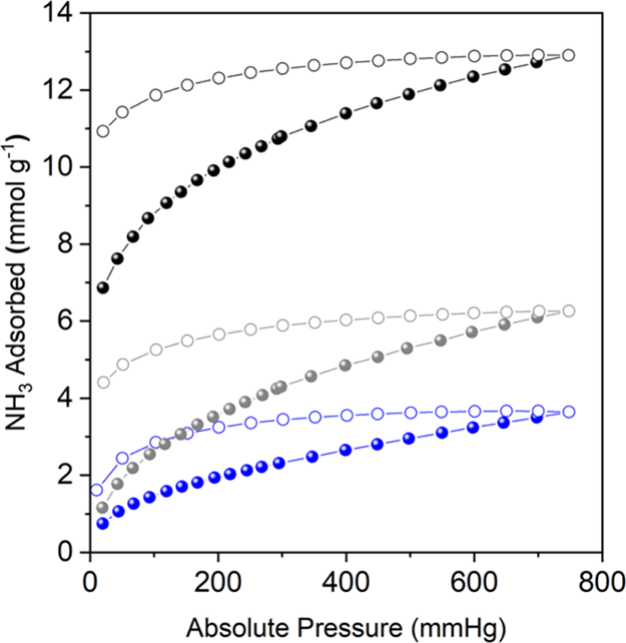
Ammonia-adsorption (solid dots) and desorption (outlined dots) at 298 K of pristine activated H-RhMOP (black), H-RhMOP after the first NH_3_-adsorption isotherm activated under vacuum (blue), and H-RhMOP after the first NH_3_-adsorption isotherm activated under vacuum and heat (130° C).

We began the ammonia sorption experiments using H-RhMOP (Figures 2 and S5). The total NH_3_ uptake at 1 bar for this MOP was 12.9 mmol/g [81.6 NH_3_ molecules per Rh-MOP; 3.4 NH_3_ molecules per Rh(II) site], which is in the range of high-performing MOFs and other extended materials (Table S1).^4,6,7,9,10,12−14,28−37^ The ammonia-uptake profile resembles a type-I isotherm and shows a steep increase in the uptake at low pressure. The desorption branch exhibited an open-loop hysteresis, in which only 26% of the adsorbed NH_3_ molecules had been desorbed at low pressure. Consequently, the performance of H-RhMOP on a second consecutive adsorption cycle dropped significantly, from the initial value of 12.9 mmol/g down to 3.6 mmol/g. Intriguingly, the uptake performance of H-RhMOP could only be partially recovered after the NH_3_-loaded sample had been heated at 130 °C under vacuum for 12 h: the resulting NH_3_ uptake at 1 bar was 6.3 mmol/g, which corresponds to 48% of the initial ammonia uptake (Figure 2).

Based on the high initial uptake of ammonia by H-RhMOP, the presence of a large hysteresis loop in the desorption branch, and the difficulty to fully regenerate H-RhMOP after loading it with ammonia, we postulated that strong chemical interactions between the MOP and the ammonia have occurred. We reasoned that the Lewis-acidic character of the Rh(II) axial sites could play an essential role in such interactions, as previously observed for other gases with electron lone pairs, such as CO and NO.^21,38^ The first evidence of the interaction of ammonia with the Rh(II) paddlewheel cluster was provided by the direct observation of the sample before and after the NH_3_ sorption experiment. The sample became purple upon adsorption, which is indicative that the coordination environment of Rh(II) is different upon NH_3_ loading, with the ammonia to be coordinated to the Rh(II) paddlewheel cluster through its N atom (Figure S6).^39^ We acquired further evidence of the Rh–N coordination through diffusive reflectance UV–vis spectra by monitoring the spectroscopic changes of the adsorption bands centered at 500–600 nm (λ_max_), which is ascribed to the π* → σ* transitions of the Rh–Rh bond, which is highly sensitive to alterations in the coordination environment of the Rh(II) axial site.^40^ Thus, the λ_max_ of H-RhMOP shifted from 598 to 540 nm upon NH_3_ adsorption, indicating that the axial site of the Rh(II) paddlewheel clusters was indeed coordinated to NH_3_ (Figure S6). However, we also reasoned that the coordination of a single NH_3_ molecule to each Rh(II) axial site alone could not explain the high amount of ammonia that was irreversibly bound to H-RhMOP, which reached 3.4 molecules of NH_3_ per Rh(II) site at 1 bar (Figure S5).

**Figure 3:**
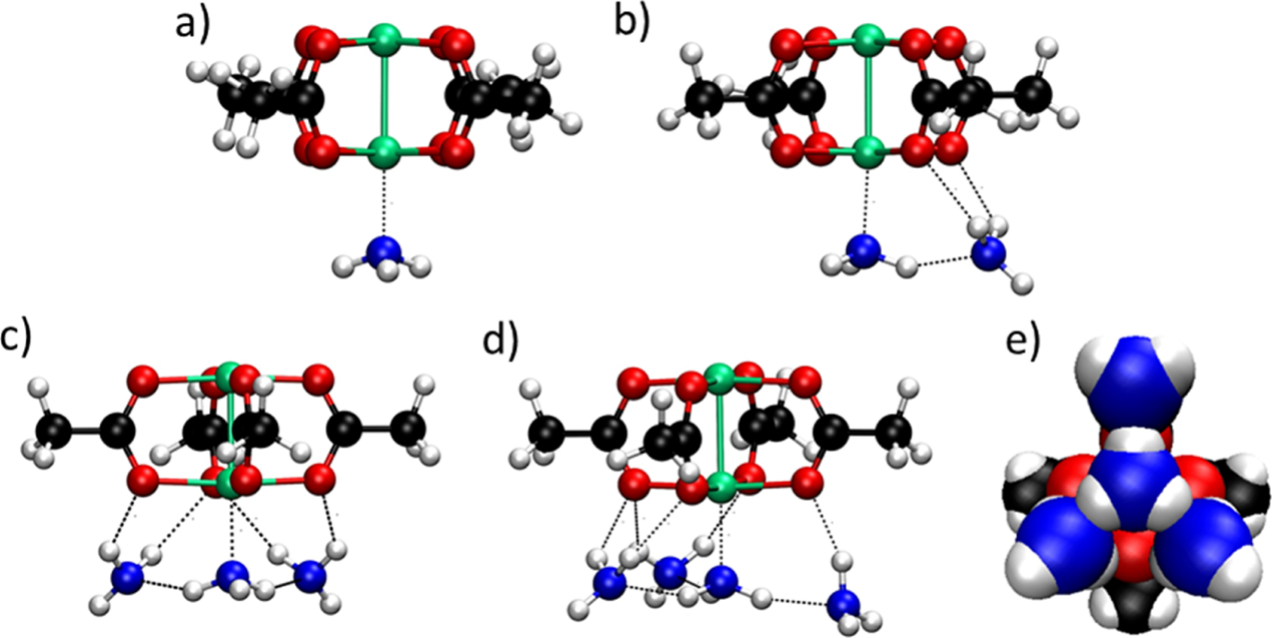
Structures obtained from DFT geometry optimizations of Rh_2_(Ac)_4_ in the presence of 1 (a), 2 (b), 3 (c), and 4 (d) molecules of NH_3_ shown in ball and stick representation. Color code: carbon (black); hydrogen (white); nitrogen (blue); and oxygen (red). The dashed lines indicate distances involved in the adsorption interactions (Rh–N: ∼2.0 Å, O···H: ∼2.0–2.3 Å, and N···H: ∼1.9–2.0 Å). (e) Top view of the same structure shown in (d) in order to appreciate the structure of the H-bond network between the four adsorbed NH_3_ molecules (atoms shown as spheres with their van der Waals size).

To elucidate the role of the Rh(II) paddlewheel cluster in the irreversible uptake of ammonia by H-RhMOP and the interactions involved in this process at the atomistic level, we have performed density functional theory (DFT) geometry optimizations and molecular dynamics (MD) simulations. For our DFT calculations, we employed Gaussian 16 at the M06-L/SDD level of theory, appropriate for the description of metal organic systems containing Rh (see the Supporting Information for details).^41,42^ Instead of the full Rh(II)-based MOPs (which is too big for DFT calculations), we have considered Rhodium acetate [Rh_2_(Ac)_4_] as a surrogate. We have calculated the optimized structures obtained after interaction of [Rh_2_(Ac)_4_] with increasing numbers of NH_3_ molecules (up to four molecules per Rh(II) site; see Figure 3). Our results show that a single NH_3_ molecule coordinates to the axial site of the Rh(II) paddlewheel cluster with a N–Rh distance of 2.2 Å and an estimated bonding energy of −31.8 kcal/mol (Figures 3a, S7 and Table S2). Subsequent addition of NH_3_ molecules to the complex is favored by approximately 10 kcal/mol, through H-bonding interactions that involve these additional NH_3_ and the oxygen atoms of the acetate ligand, with O···H distance of ∼2.2 Å (Figures 3 and S7). The adsorption of up to four NH_3_ molecules per Rh(II) site is possible due to the formation of a coordination-assisted H-bonding network of NH_3_ molecules: one NH_3_ molecule is coordinated to the axial Rh(II) site, and it is surrounded by three H-bonded NH_3_ molecules that are 120° apart from each other with N···H distance of ∼2.0 Å (Figures 3e and S8). It has to be noted that the capacity of ammonia to form such H-bonded ammonia clusters in the liquid and solid state has been previously described through computational and experimental methods.^43,44^ In our case, the initial Rh(II)-coordinated NH_3_ molecule is what serves as the seed to nucleate the formation of such clusters. Remarkably, DFT calculations showed that this initial Rh(II)–NH_3_ coordination occurs even in the presence of water, highlighting the high affinity of Rh(II) paddlewheel clusters toward ammonia (Figure S9). Overall, DFT calculations did not reveal any destabilization, breakage, or desymmetrization of Rh_2_(Ac)_4_ upon addition of ammonia, thus further corroborating the high chemical stability of this metal–organic complex.

**Figure 4:**
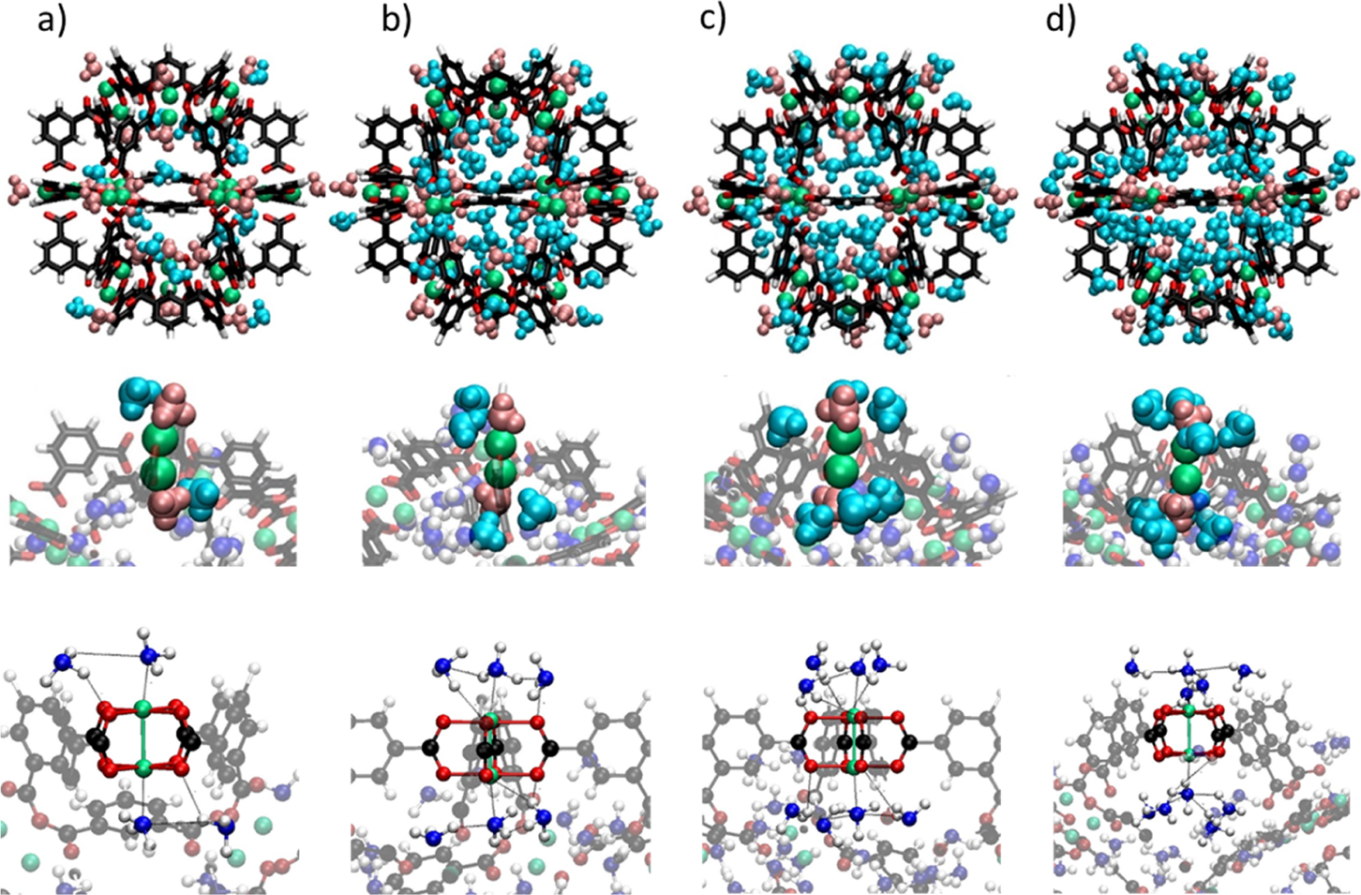
Results from MD simulation of a H-RhMOP in the presence of different amounts of NH_3_ at 298 K. Top row: screenshots of the instantaneous configurations obtained after equilibration in a simulation box of 11.4 (a), 28.4 (b), 45.7 (c), and 57.1 molecules/nm^3^ (d). For simplicity, only the NH_3_ molecules closer to the H-RhMOP are shown. The NH_3_ molecules and the Rh atoms are shown with its van der Waals size, and the rest of the H-RhMOP structure is shown as balls and sticks. Color code: coordinated NH_3_ molecules (pink); H-bonded NH_3_ molecules (cyan); and rhodium (green). Middle row: zoomed-in images of the corresponding environments of the Rh(II) paddlewheel cluster in each simulation experiment. Bottom row: detailed chemical environment of the Rh(II) paddlewheel cluster for each of the simulated systems.

Next, we employed the results from DFT calculations to parametrize appropriate force fields (see the Supporting Information) for performing MD simulations between the entire H-RhMOP and NH_3_ molecules. The simulations were designed, performed, and analyzed using NAMD and VMD software (see full details of the simulations in the Supporting Information).^45,46^ Briefly, the simulated systems consisted of a H-RhMOP placed in the middle of a simulation box containing a fixed amount of NH_3_ molecules in the gas phase, thermostated at 298 K. We considered simulations with the same amount of molecules but different volumes, corresponding to different gas densities and consequently mimicking different points of the experimental adsorption isotherms. We observed that even for those simulations with the largest volume (lowest gas density), each Rh(II) axial site was occupied with one coordinated NH_3_ molecule (Figures 4a and S10). Additionally, 60% of the Rh(II) paddlewheel clusters contained one additional NH_3_ molecule, which was stabilized through H-bonding interactions (Table S5). Thus, at low ammonia pressure, the average number of NH_3_ per Rh(II) site was 1.6. This result agrees with the experimental value of 1.5 NH_3_ per Rh(II) site that we had previously found at low ammonia pressure (19.7 mmHg, 0.03 bar) (Figure S5). Further shrinking of the simulation box induced adsorption of more NH_3_ molecules to the Rh(II) sites, via H-bonding interactions with the already-coordinated NH_3_ molecule and with the O atom of the carboxylic group of the BDC ligand, until an equilibrium configuration of *ca.* four NH_3_ molecules per Rh(II) site was reached (Figure 4b–d and Table S5). Interestingly, using Fourier transform infrared (FTIR) spectroscopy, we found experimental evidence that the carboxylic group of the BDC ligand participates in the H-bonding network of the NH_3_ molecules. The carboxylic region (C=O_str_) of the FTIR spectrum of the NH_3_-loaded H-RhMOP was shifted by *ca.* 12 cm^–1^ relative to the pristine sample, a value compatible with the O atoms of the carboxylic groups contributing to formation of the H bonds (Figure S12), as predicted by our simulations.^12^

The results of the DFT calculations and MD simulations indicate that adsorption of ammonia in H-RhMOP entails a two-step mechanism that involves two different interactions. The first step, occurring at low ammonia pressure, involves the coordination of one NH_3_ molecule to both axial sites of the Rh(II) paddlewheel clusters of Rh-MOP. In the second step, a NH_3_ molecule coordinated with Rh(II) nucleates the adsorption of additional NH_3_ molecules, which are stabilized through two types of H-bonds: N–H···H interactions between coordinated and non-coordinated NH_3_ and N–H···O=C interactions between non-coordinated NH_3_ and BDC linkers. This second steps occurs at higher NH_3_ densities and consequently at higher NH_3_ pressure. This mechanism would be responsible for the uptake of up to four molecules of NH_3_ per Rh(II) site, as seen in our MD simulations, which translates into a theoretical adsorption capacity of 15.0 mmol/g for H-RhMOP—a value that is close to the experimental value of 12.9 mmol/g. The high stabilizing energies predicted by our DFT calculations justify the irreversible adsorption of NH_3_ in H-RhMOP experimentally found in the NH_3_-adsorption/desorption isotherms.

Based on the values above, we concluded that the uptake of ammonia by H-RhMOP is dominated by the chemistry of the Rh(II) paddlewheel cluster and does not involve any significant contribution of physisorbed NH_3_ molecules within the cavities of H-RhMOP. We pondered whether the high ammonia uptake in H-RhMOP could be achieved without structuring Rh(II) paddlewheel clusters in a porous cage. To test this hypothesis, we checked the ammonia-adsorption uptake capacity of Rh_2_(Ac)_4_. Interestingly, we found that Rh_2_(Ac)_4_ could adsorb 1.5 mmol/g of NH_3_ [0.3 NH_3_ molecules per Rh(II) site] at 1 bar, which we attributed to the lack of diffusion pathways throughout the sample, as this would disfavor interactions between NH_3_ molecules and the potentially available Rh(II) sites (Figure S13). Furthermore, we reasoned that close packing between adjacent Rh_2_(Ac)_4_ in the solid state would not leave any space to establish the H-bonding network of the NH_3_ molecules organized around the coordinated NH_3_. Therefore, we concluded that cavities and diffusion pathways are essential to generate the coordination-induced H-bonding network of NH_3_ molecules at the Rh(II) axial sites.

### 2.2 Regeneration of H-RhMOP

We targeted the regeneration of the Rh(II) axial sites of H-RhMOP through a chemical method based on a ligand-exchange reaction with water, which benefits from the high hydrolytic stability of Rh-MOPs, even at low pH.^47^ Thus, NH_3_-loaded H-RhMOP was immersed in acidic water (pH = 2) for 5 min, after which the sample changed from purple to green, indicating that the ligand at the axial site of the Rh(II) paddlewheel cluster had exchanged from an N-donor (i.e., NH_3_) to an O-donor (i.e., H_2_O) (Figure S14). Low pH accelerates this process as it promotes the formation of non-coordinating NH_4_^+^ cations. Evidence of ligand exchange was acquired from diffusive reflectance UV–vis spectra. The spectrum obtained after incubating the NH_3_-loaded H-RhMOP in acidic water revealed a λ_max_ of 593 nm, which confirmed the release of the NH_3_ molecule from the axial site of Rh(II) (Figure S6).

**Figure 5:**
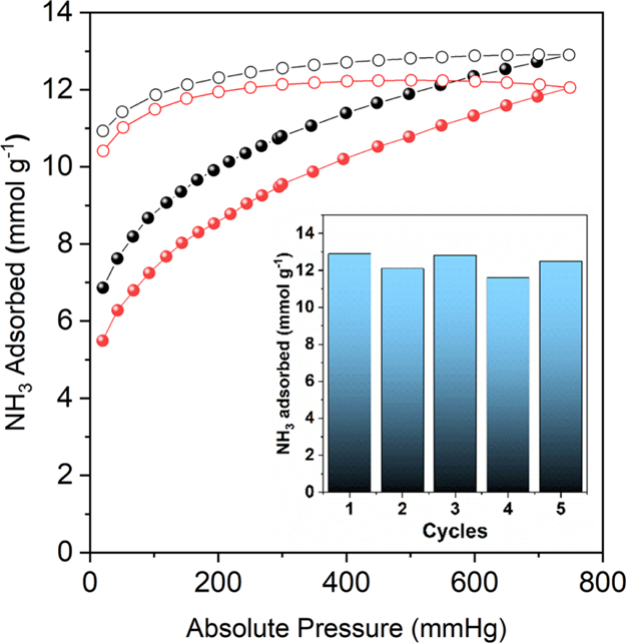
Ammonia-adsorption (solid dots) and desorption (outlined dots) at 298 K of pristine activated H-RhMOP (black) and regenerated H-RhMOP (red). Inset: ammonia uptake of H-RhMOP at 1 bar and 298 K for each of the five successive ammonia-adsorption/aqueous-regeneration cycles.

Once the axial site of the H-RhMOP was regenerated, we investigated the efficiency of the regeneration process, by measuring the second cycle of ammonia adsorption. To this end, the activated H-RhMOP was heated at 130 °C under vacuum for 12 h, and the NH_3_-adsorption isotherm was collected. As depicted in Figure 5, the ammonia uptake of the regenerated sample was 12.1 mmol/g, which corresponds to an overall regeneration efficiency of 94%. This chemical and thermal regeneration method was repeated for five consecutive cycles, and no significant loss in the ammonia-uptake capacity of H-RhMOP was observed (Figure 5, inset).

### 2.3 Ammonia Adsorption in Surface-Functionalized Rh-MOPs

**Figure 6:**
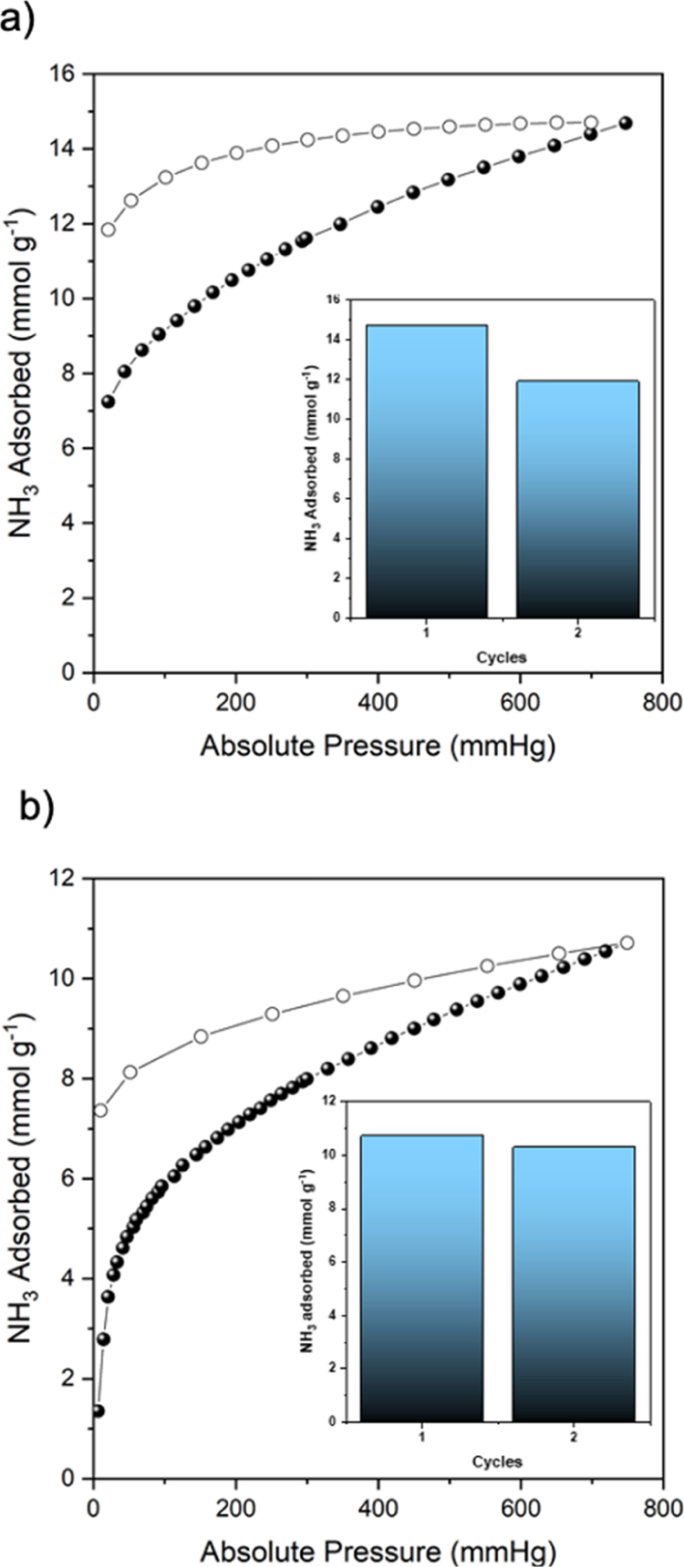
(a) Ammonia-adsorption (solid dots) and desorption (outlined dots) at 298 K of pristine activated OH-RhMOP. Inset: ammonia uptake of OH-RhMOP at 1 bar and 298 K for the two successive NH_3_-adsorption/aqueous–regeneration cycles. (b) Ammonia-adsorption (solid dots) and desorption (outlined dots) at 298 K of pristine activated C_12_-RhMOP. Inset: ammonia uptake of C_12_-RhMOP at 1 bar and 298 K for the two successive NH_3_-adsorption/aqueous–regeneration cycles.

Based on our proposed mechanism by which the basic cuboctahedral H-RhMOP unit interacts with NH_3_ molecules, we then evaluated the impact of surface functionality on ammonia uptake by measuring the NH_3_ sorption of two differently surface-functionalized Rh-MOPs: OH-RhMOP and C_12_-RhMOP. As depicted in Figure 6, OH-RhMOP and C_12_-RhMOP each exhibited a type-I adsorption isotherm, with maximum values for ammonia uptake (at 1 bar) of 14.7 mmol/g [100.8 NH_3_ molecules per Rh-MOP/4.2 NH_3_ molecules per Rh(II) site] and 10.7 mmol/g [115.2 NH_3_ molecules per Rh-MOP/4.8 NH_3_ molecules per Rh(II) site], respectively. Moreover, both functionalized Rh-MOPs presented a large, open-loop hysteresis in the corresponding desorption branch. As with H-RhMOP, the performance could only be recovered after the water and thermal treatments (Figures S15 and S16). The overall regeneration efficiencies were 83% for OH-RhMOP and 96% for C_12_-RhMOP (Figure 6). We ascribed the lower recovery efficiency of OH-RhMOP to the presence of acidic hydroxyl protons at the surface, which can protonate ammonia to generate ammonium phenolate, which could be more challenging to regenerate compared to H-bonded and Rh(II)-coordinated NH_3_ molecules.^32,48,49^ Matrix-assisted laser desorption ionization time-of-flight measurements of both functionalized Rh-MOPs enabled confirmation of the integrity of these Rh-MOPs after two consecutive NH_3_-adsorption/aqueous-regeneration cycles (Figure S17). Interestingly, the lower BET surface areas (*S*_BET_) of OH-RhMOP (*S*_BET_ = 480 m^2^/g) and of C_12_-RhMOP (non-porous to N_2_) relative to H-RhMOP (*S*_BET_ = 940 m^2^/g) do not correlate to the ammonia-uptake capacity of these materials. Conversely, the ammonia uptake for both OH-RhMOP (4.2 NH_3_ molecules per Rh(II) site) and C_12_-RhMOP (4.8 NH_3_ molecules per Rh(II) site) exceeded that for H-RhMOP (3.4 NH_3_ molecules per Rh(II) site. We attributed this difference to the presence of available H-donors (hydroxyl groups, in OH-RhMOP) or H-acceptors (ether groups, in C_12_-RhMOP) at the periphery of the functionalized Rh-MOPs. Thus, we concluded that the uptake of functionalized Rh-MOPs could be increased via peripheral H-bonding interactions between the surface functional groups and NH_3_ molecules. This interpretation was supported by additional MD simulations performed for functionalized Rh-MOPs (Figures S18 and S19). We found that the activities of the Rh(II) sites in OH-RhMOP and in C_12_-RhMOP are equivalent to that which we found for H-RhMOP. Thus, each Rh(II) site can take up to four molecules of NH_3_, through the coordination-induced H-bonding mechanism described above. Furthermore, functionalized Rh-MOPs showed additional H-bonding interactions between their external surface groups and their adsorbed NH_3_ molecules, which would account for their superior ammonia-uptake relative to H-RhMOP (Figures S20 and S21). Overall, these results coincide with the observed trend in MOFs containing open-metal sites, in which the interaction strength of binding sites is a better predictor of ammonia uptake than is the surface area.^10,13^

## 3 Conclusions

In summary, we have reported the first-ever study on MOPs for the uptake of gaseous ammonia. We investigated three isostructural cuboctahedral Rh-MOPs with different surface functionalities, all interacting strongly with NH_3_ molecules through their Rh(II) axial sites. We unveiled the mechanism that underpins the interaction between Rh-MOPs and NH_3_ through experiments and simulations, finding an NH_3_ molecule that initially interacts with the Rh(II) paddlewheel cluster by coordinating with their axial sites. The coordinated NH_3_ molecule then templates the subsequent addition of NH_3_ molecules, which are stabilized through H-bonding interactions. This coordination-induced H-bonding network of NH_3_ on top of each Rh(II) axial site increases the number of NH_3_ molecules that can be taken up per Rh(II) paddlewheel cluster, which, at the saturation point, reaches eight NH_3_ molecules per Rh(II) paddlewheel cluster. The coordination-induced NH_3_-cluster formation that we have described here is consistent with previously postulated ammonia-adsorption mechanisms for some of the best-performing MOFs such as M_2_Cl_2_BBTA [where M is Co(II), Ni(II), and Cu(II) and BTA is 1*H*,5*H*-benzo(1,2-*d*),(4,5-*d*′)bistriazole]^50^ and MFM-300 MOF.^30^ However, those MOFs relay on high-nuclearity clusters to trigger the formation of NH_3_ clusters, Rh-MOPs instead depend on low-nuclearity Rh(II) paddlewheel clusters as NH_3_-binding sites, thus highlighting the uniqueness of this class of MOPs. Furthermore, we discovered that the overall ammonia-uptake efficiency of Rh-MOPs could be further enhanced by the judicious use of surface functional groups that can establish H-bonding interactions, such as hydroxyls (as H-donors) or ethers (as H-acceptors). Overall, our results demonstrate the viability of using discrete molecular cages as porous hosts for capture of ammonia. We believe that the capacity of MOPs to be processed into materials such as films,^51^ gels, and monoliths,^52^ or even porous solutions^53^ should contribute to developing a new class of shaped adsorbents for ammonia.

## Supporting Information Available

Detailed experimental procedures, material characterization, methods, and ammonia isotherms (PDF)
