# Supplementary material for: Ammonia Capture in Rhodium(II)-Based Metal–Organic Polyhedra via Synergistic Coordinative and H-Bonding Interactions
Source: ACS Appl Mater Interfaces. 2023 Jan 25;15(5):6747–54. doi: 10.1021/acsami.2c19206 (PMC9923682; doi:10.1021/acsami.2c19206)
Supplement: Supplementary file 1 — am2c19206_si_001.pdf [file am2c19206_si_001.pdf]

Supporting Information

Ammonia Capture in Rhodium(II)-based Metal-organic Polyhedra via Synergistic Coordinative and H-bonding Interactions

Arnau Carné-Sánchez,[a,b*] Jordi Martínez-Esaín,[a] Tanner Rookard,[c] Christopher J. Flood,[c] Jordi Faraudo,[d] Kyriakos C. Stylianou[c*] and Daniel Maspoch[a,b,e*]

[a] Catalan Institute of Nanoscience and Nanotechnology (ICN2), CSIC, and Barcelona Institute of Science and Technology, Campus UAB, 08193 Bellaterra, Barcelona, Spain

Email: arnau.carne@icn2.cat; daniel.maspoch@icn2.cat

[b] Departament de Química, Facultat de Ciències, Universitat Autònoma de Barcelona, 08193 Bellaterra, Spain

[c] Institut de Ciència de Materials de Barcelona (ICMAB-CSIC), 08193 Bellaterra, Spain

[d] Department of Chemistry, Oregon State University, Corvallis, OR 97331-4003, USA

[e] ICREA, Pg. Lluís Companys 23, 08010 Barcelona, Spain

Table of Contents

S1. Materials and experimental methods.

S1.1 Materials.

S1.2 Experimental methods.

S2. Computational methods

S3. Characterization of Rh-MOPs used as adsorbents for NH_3

S4. NH_3 uptake in H-RhMOP

S5. Computer simulation of the interaction between H-RhMOP and NH_3

S5.1. DFT calculations of the interaction between $Rh_2(Ac)_4$ and NH_3

S5.1.2. DFT calculations of the interaction between $Rh_2(Ac)_4$, NH_3 and H_2O

S5.2. Computer simulation of the interaction between H-RhMMOP and NH_3

S5.2.1. Parametrization of the Force Field from DFT calculations

S5.2.2. Molecular dynamic simulations of the interaction between H-RhMOP and NH_3

S6. FTIR spectroscopy of ammonia-loaded H-RhMOP

S7. NH_3 uptake in $Rh_2(Ac)_4$

S8. Digital photographs showing the regeneration of H-RhMOP

S9. NH_3 uptake in OH-RhMOP and C_{12}-RhMOP

S10. Computer simulation of the interaction between functionalized Rh-MOPs and NH_3

S10.1 OH-RhMOP and NH_3

S10.2 C_{12}-RhMOP and NH_3

S1. Materials and Experimental Methods.

S1.1 Materials

Rhodium acetate was purchased from Acros Organics. Benzenedicarboxylic acid, 5-hydroxy-1,3-benzenedicarboxylic acid, dimethyl 5-hydroxyisophthalate, 1-bromododecane and sodium carbonate were purchased from Sigma-Aldrich. All deuterated solvents were purchased from Eurisotop. Solvents at HPLC grade were purchased from Fischer Chemicals.

Nitrogen adsorption measurements were collected at 77 K using an ASAP 2460 (Micromeritics). Temperature for N_2 isotherms measurement was controlled by using a liquid nitrogen bath.

Mass Spectroscopy (MALDI-TOF) measurements were performed using a 4800 PlusMALDI TOF/TOF (ABSCIEX – 2010). The matrix used in each case were: trans-2-[3-(4-*tert*-butylphenyl)-2-methyl-2-propenylidene]malononitrile (DCTB) for C_{12}RhMOP, measured in positive mode; and sinapinic acid for ONaRhMOP, measured in negative mode.

Powder X-ray diffraction (PXRD) diagrams were collected on a Panalytical X'pert diffractometer with monochromatic Cu-Kα radiation (λ_{Cu} = 1.5406 Å). Samples were measured in a capillary.

Proton nuclear magnetic resonance (^{1}H-NMR) spectra were acquired in a Bruker Ascend 300 MHz and a Bruker Avance III 400SB NMR spectrometer at "Servei d'Anális Química" from Autonomous University of Barcelona (UAB).

Ultraviolet-Visible Spectrophotometry (UV-Vis) spectra were collected with a PerkinElmer Lambda 1050+ spectrometer from 300-800 nm with a step width of 1.5 nm using materials in the solid state.

NH_3 Adsorption−Desorption Isotherms were conducted on a Micromeritics 3Flex at 298K and to an absolute pressure of ammonia (purity 99.999) of about 750 mmHg (1 bar). Temperature was controlled with Micromeritics ISO Controller. Before these experiments were conducted, the materials were activated (guest and solvent molecules were removed from the pores) by heating at 120ºC under vacuum for 12 hours.

Thermogravimetric Analysis. TGA curves were collected on a TA Instruments SDT Q600 under argon from ambient temperature to 500°C with a ramp of 5.0 °C/min.

S1.2 Experimental Methods

5-dodecoxybenzene-1,3-dicarboxylic acid H (C_{12}O-bdc) was synthesized following a previously described protocol.[1]

H-RhMOP was synthesized following a previously described protocol.[2] The as-made sample was exchanged with acetone for 5 days before gas adsorption measurements.

OH-RhMOP was synthesized following a previously described protocol.[3] OH-RhMOP was exchanged with a diethyl ether : acetone mixture (1:1 vol%) for 5 days before gas sorption measurements.

C_{12}-RhMOP was synthesized following a previously described protocol.[4] The as-made sample was exchanged with ethanol for 5 days before gas adsorption measurements.

S2. Computational methods

Density Functional Theory Calculations. All calculations were carried out using the Gaussian 16 program revision B.01.[5] We employed density functional theory (DFT) method at the M06-L/SDD level of theory. We selected the M06-L hybrid functional of Truhlar and Zhao because of its broad accuracy for metallochemical calculations for all metals.[6] Previous calculations of metalorganic paddlewheel structures in vacuo comparing different DFT functionals and basis sets with MP2 ab initio quantum chemistry calculations showed that MP06 is the best performing functional irrespective of the basis set employed (energies and geometrical quantities such as bond distances and angles calculated with this functional show little dependence on the specific basis set employed).[7] For our calculations, we selected the commonly used SDD basis set because it is suitable for both Rh(II) and acetate. It combines double zeta with the Stuttgart-Dresden ECP, which reduces the cost caused by a large number of electrons, giving close agreement with experimental results.[8]

Molecular Dynamics Simulations. All MD simulations were performed using the NAMD program, version 1.14.[9] The species included in the simulations are ammonia molecules in gas phase and a single Rh-MOP (H-RhMOP, OH-RhMOP or OMe-RhMOP) fixed in the center of the simulation box. In order to simplify the calculation, the atomic positions of the MOP were fixed during the simulations and only NH_3 molecules were allowed to move. The force field employed in the simulation was a modification of the CHARMM force field to include Rh interactions as determined by our DFT calculations, as described below. The initial structures for the simulations were prepared as follows. First, we start building the atomic coordinates of the Rh-MOPs based on the experimental single crystal crystallographic information.[2] The structures were $[Rh_2(H\text{-}bdc)_2]_{12}$, $[Rh_2(OH\text{-}bdc)_2]_{12}$ and $[Rh_2(OMe\text{-}bdc)_2]_{12}$ for the cases of H-RhMOP, OH-RhMOP and OMe-RhMOP respectively (where H-bdc is 1,3-benzenedicarboxylate, OH-bdc is 5-hydroxy-1,3-benzenedicarboxylate and OMe-bdc is 5-methoxy-1,3-benzenedicarboxylate). Then, ammonia molecules were introduced randomly distributed around the Rh-MOPs without contact. We considered several simulations with different simulation box sizes in order to model different NH_3 densities which correspond to different points of the adsorption isotherm. In all cases, before running the actual MD simulations, we performed an energy minimization to correct any possible bad contacts between atoms. In the MD simulations we solved the Newton equations of motion using a time step of 2 fs. Electrostatic interactions were computed using the PME method with the standard settings in NAMD (1 Å resolution, updated each 2 time steps). Lennard-Jones interactions were truncated at 1.2 nm employing a switching function starting at 1.0 nm. Periodic boundary conditions were employed in all directions. The temperature was fixed at 298 K using the Langevin thermostat (relaxation time 1 ps). The simulations were performed for times between 10-50 ns time depending on each case. The simulation times were selected monitoring the number of adsorbed molecules and ensuring that this quantity has equilibrated.

S3. Characterization of Rh-MOPs used as adsorbents for NH_3

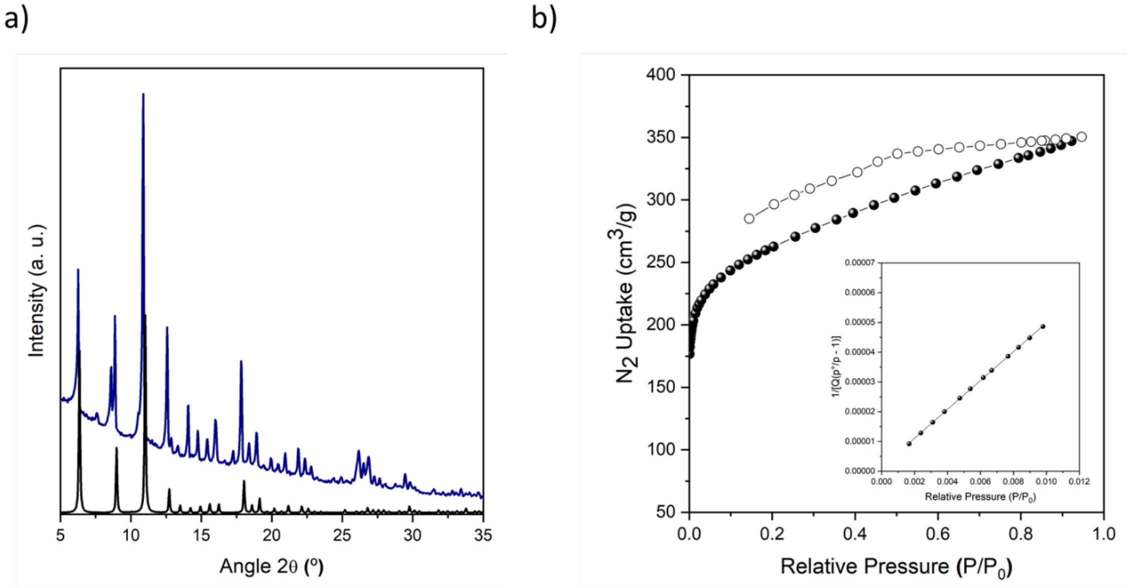

Figure S1. (a) Simulated PXRD diffractogram of H-RhMOP from crystal structure (black) and experimental PXRD diffractogram of as-made crystals of H-RhMOP. (b) N_2 adsorption-desorption isotherm for H-RhMOP at 77 K (S_{BET} = 940 m^2/g).

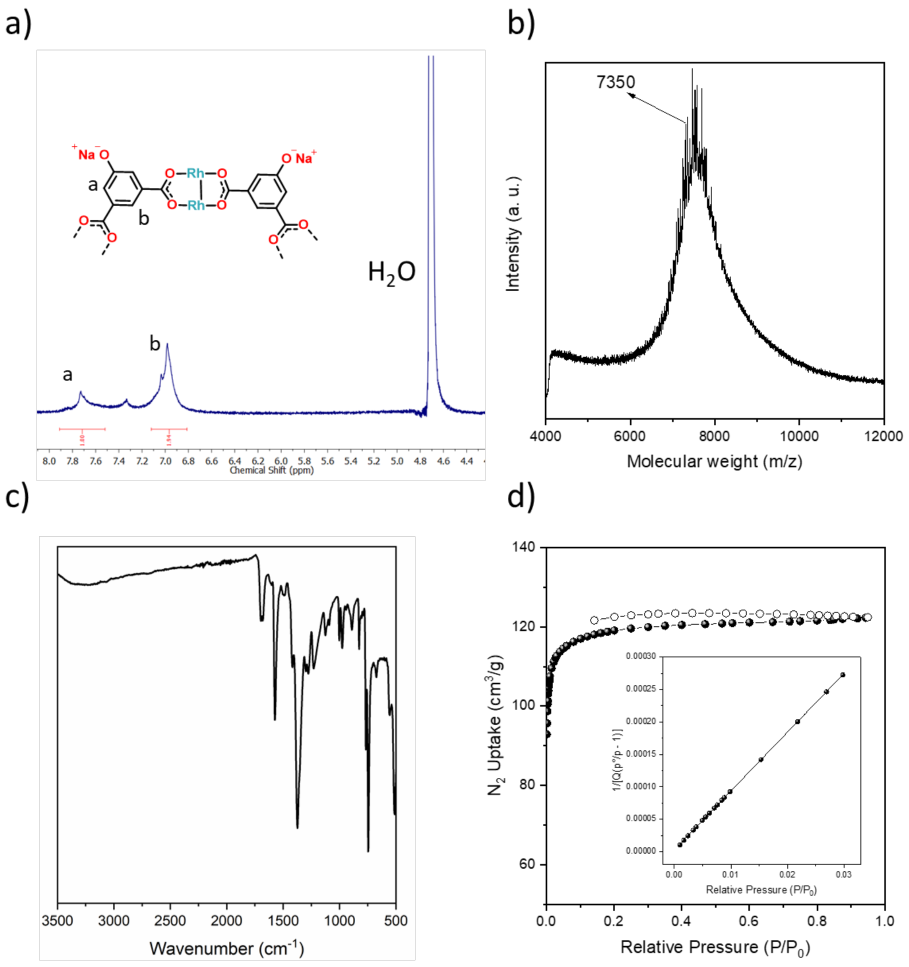

Figure S2. (a) ^{1}H-NMR (300 MHz, D_2O) spectrum of OH-RhMOP solubilized in basic water (pD = 12). (b) MALDI-TOF spectrum of OH-RhMOP solubilized in basic water (pH = 12). The weight corresponding to the formula $[Rh_{24}(OBDC)_{24} + 23Na]^-$ + MeOH has been highlighted: expected = 7352; found = 7350. (c) FTIR spectrum of OH-RhMOP. (d) N_2 adsorption-desorption isotherm for OH-RhMOP at 77 K (S_{BET} = 480 m^2/g).

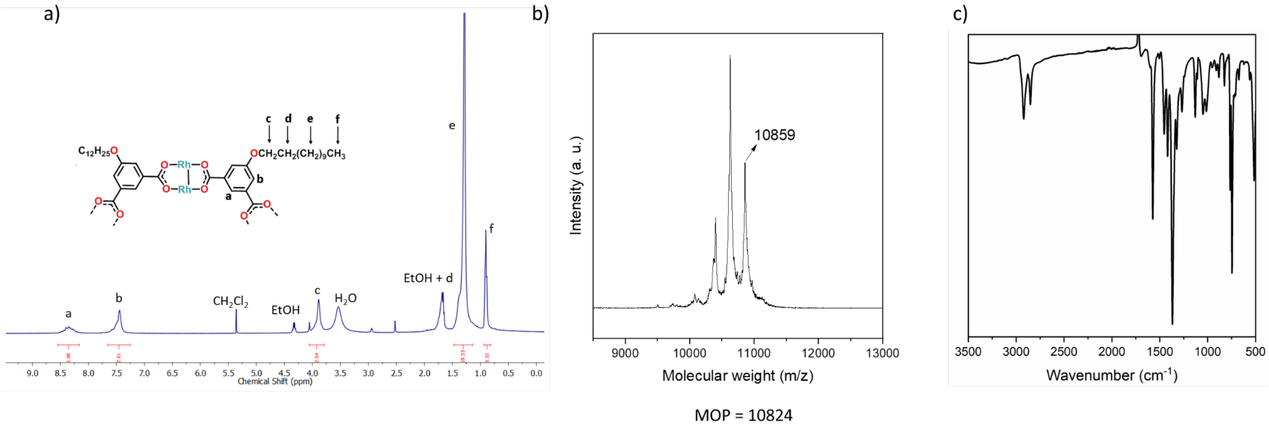

Figure S3. (a) ^{1}H-NMR (300 MHz, CD_2Cl_2) spectrum of C_{12}-RhMOP. (b) MALDI-TOF spectrum of C_{12}-RhMOP. The weight corresponding to the formula $[Rh_{24}(C_{12}O\text{-}BDC)_{24} + H]^+ + 2H_2O$ has been highlighted: expected = 10858; found = 10859. (c) FTIR spectrum of C_{12}-RhMOP.

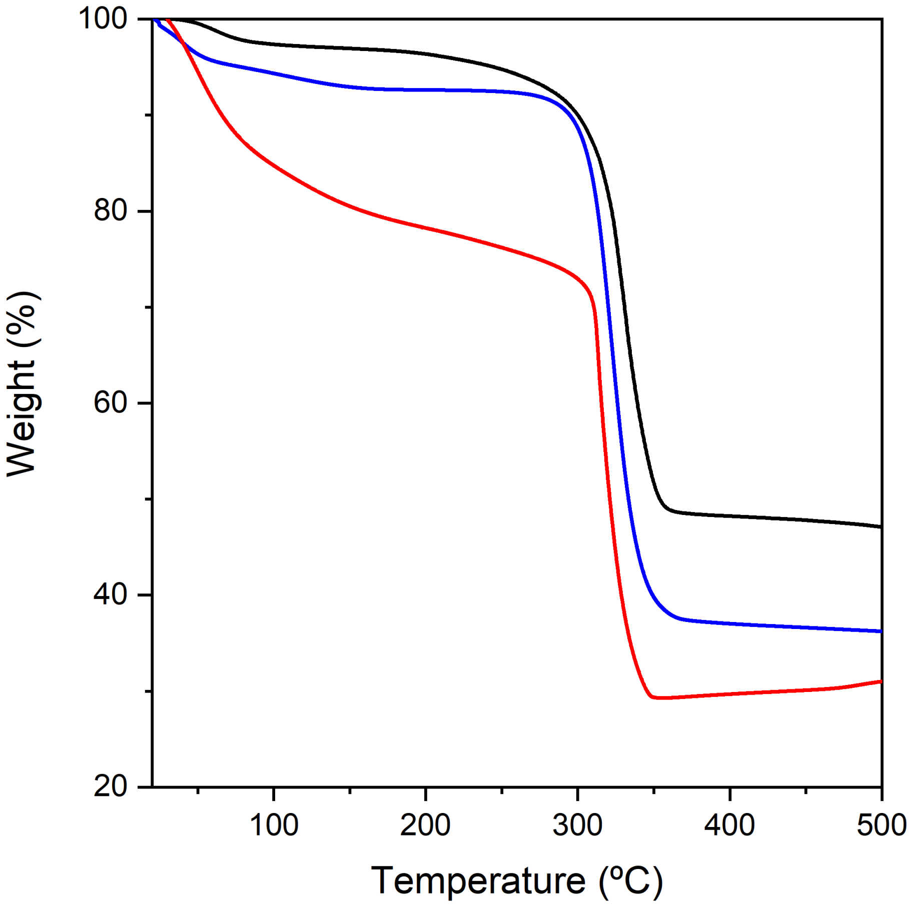

Figure S4. Thermogravimetric analysis (TGA) of H-RhMOP (black), C_{12}-RhMOP (blue) and OH-RhMOP (red). Note that OH-RhMOP shows a higher percentage of weight loss before decomposition (*ca.* 300ºC). This is attributed to its highly hydrophilic character that makes it uptake atmospheric water before the TGA measurement.

S4. Ammonia uptake in H-RhMOP

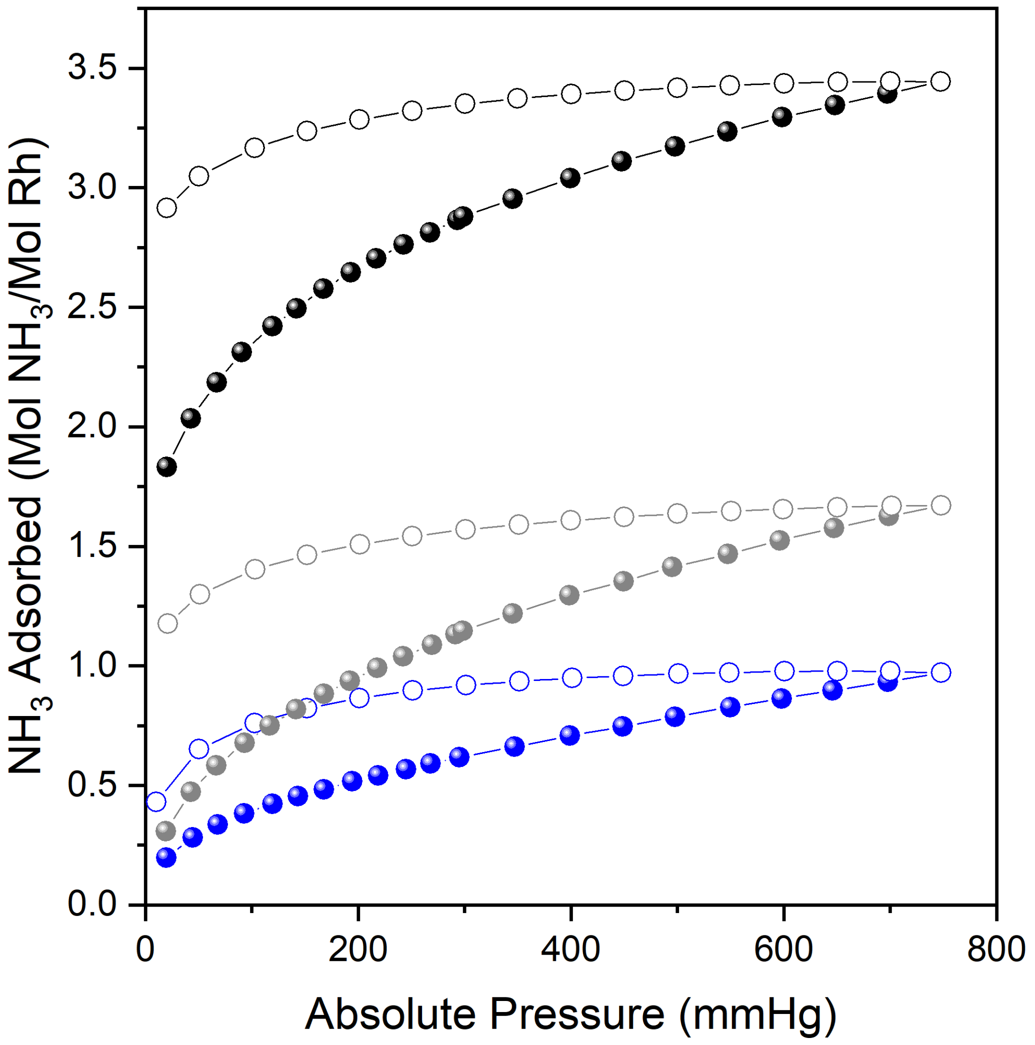

Figure S5. Ammonia-adsorption (solid dots) and -desorption (outlined dots) at 298 K of pristine activated H-RhMOP (black); H-RhMOP after the first NH_3-adsorption isotherm activated under vacuum (blue); and H-RhMOP after the first NH_3-adsorption isotherm activated under vacuum and heat (130º C). The NH_3 uptake was normalized per mol of Rh(II) site in H-RhMOP. For this calculation, the molecular weight of H-RhMOP (6405 g/mol) was considered.

Table S1. Ammonia uptake of several selected robust porous materials.

Compound	Type of material	NH_3 adsorbed at 1 bar and 298K (mmol/g)	Reference
	**Metal-organic frameworks (MOFs)**		
Ni_Acryl_TGA		23.5	[10]
Cu_2Cl_2BBTA		19.8	[11]
Co_2Cl_2BBTA		17.8	[11]
Fe-MIL-SO_3H		17.8	[12]
MFU-4		17.7	[13]
Cu(BDC)		17.1	[14]
MFM(Al)		15.7	[15]
MFM(Sc)		13.1	[16]
UiO-66-NH_3Cl		12.0	[12]
Ga-PMOF		10.5	[17]
UiO-66-NH_2		9.8	[12]
NU-1000-F-120		7.9	[18]
	**Porous organic polymers (POPs) & Covalent-organic frameworks (COFs)**		
P1-PO_3H_2	POP	18.7	[19]
BPP-5	POP	17.7	[12]
TpBD-$(SO_3H)_2$	COF	11.5	[20]
$[HOOC]_{17}$-COF	COF	9.34	[21]
PIP-X	POP	8.8	[22]
1TCS	POP	8.52	[23]
	**Inorganic porous materials**		
NPC-PEG-AC	Nanoporous Carbon	17.0	[24]
13X zeolite WE894	Zeolite	9.3	[25]
NaP	Zeolite	8.5	[26]
MPTS-1.0	Silica Gel	7.0	[27]
	**Metal-Organic Polyhedra (MOPs)**		
H-RhMOP		12.9	This Work
OH-RhMOP		14.7	This Work
C_{12}-RhMOP		10.7	This Work

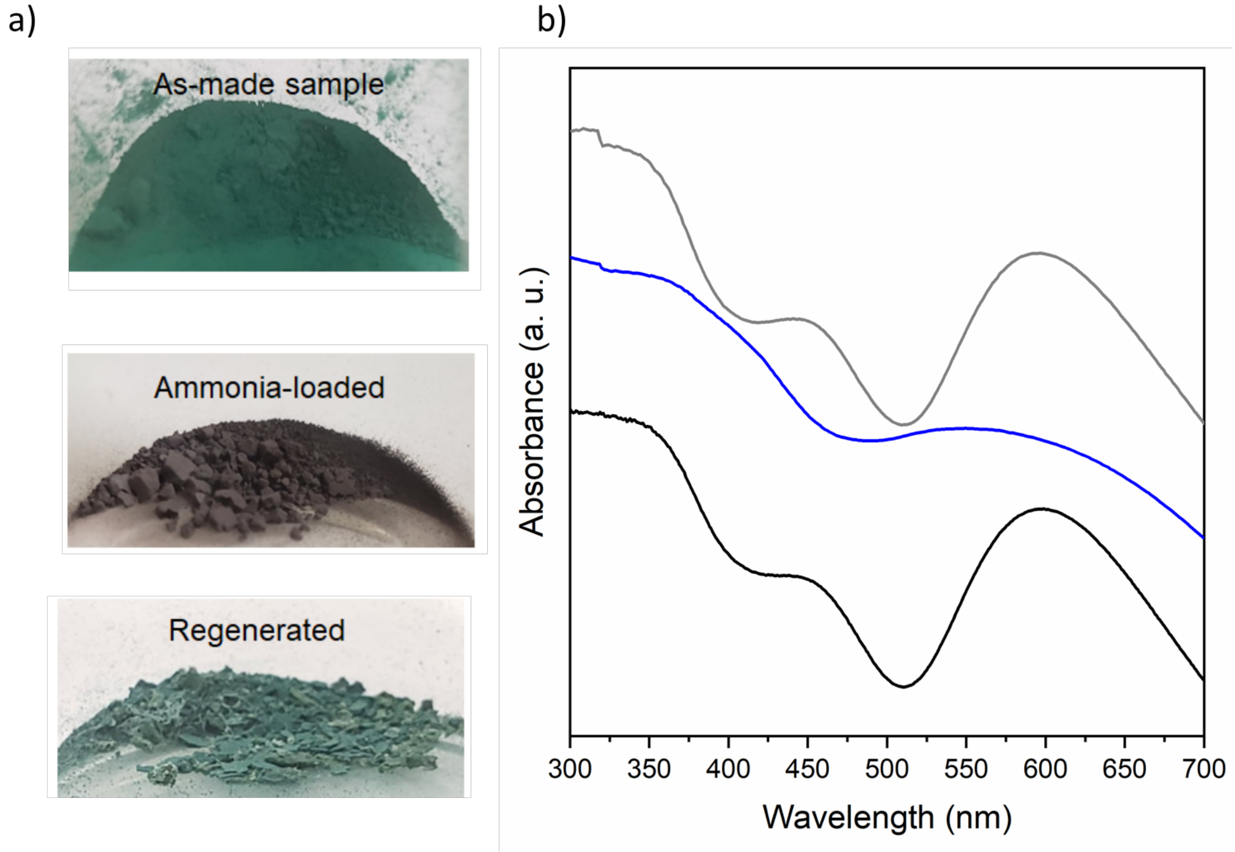

Figure S6. (a) Photographs of as-made H-RhMOP (top), H-RhMOP after NH_3 adsorption-desorption isotherm (middle), and H-RhMOP after the regeneration process (bottom). (b) Solid-state UV-Vis spectra of as-made H-RhMOP (black) (λ_{max} = 598 nm), H-RhMOP after NH_3 adsorption-desorption isotherm (blue) (λ_{max} = 540 nm), and H-RhMOP after the regeneration process (λ_{max} = 598 nm) (grey). Note that the presence of both of the characteristic bands of the Rh(II)-paddlewheel [Band I, λ_{max} = 600 - 540 nm) and Band II (λ_{max} = 370 - 450 nm)] are preserved through the NH_3 adsorption-desorption isotherms and the water-based regeneration process. These results highlight the integrity of H-RhMOP.

S5. Computer simulation of the interaction between H-RhMOP and NH_3

S5.1. DFT calculations of the interaction between $Rh_2(Ac)_4$ and NH_3

Instead of the full Rh(II) paddlewheel cluster (which is too big for DFT calculations), we considered rhodium acetate [$Rh_2(Ac)_4$] as a surrogate and different numbers of NH_3 molecules. We performed a geometry optimization in 4 different cases, corresponding to [$Rh_2(Ac)_4$] and 1, 2, 3 and 4 NH_3 molecules, as seen in **Figure S6**.

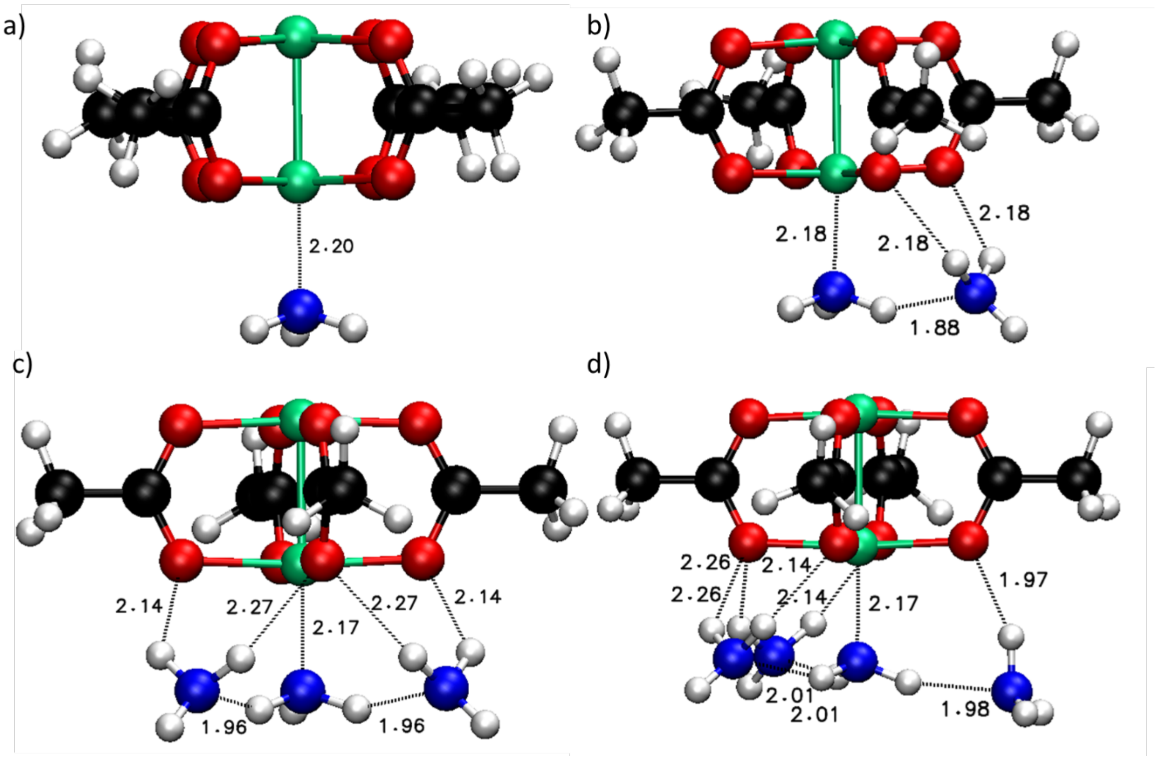

Figure S7. Optimized structures obtained in DFT calculations containing 1 (a), 2 (b), 3 (c) and 4 (d) NH_3 molecules per Rh(II) paddlewheel cluster. All distances are given in Å.

The optimized structures obtained for the four different simulations showed that Rh(II)-paddlewheel can interact with ammonia through two different interactions: coordinative (1 NH_3 molecule per Rh(II) site) and through H-bonding (up to 3 NH_3 molecules per Rh(II) site). The binding energy of the Rh-NH_3 coordination bond was found as follows:

(Eq. 1) $E(Rh\text{-}N) = E(PW\_NH_3) - \{E(PW) + E(NH_3)\}$ (where PW is Rh(II)-paddlewheel)

Using Equation 1, we obtain a value of -31.75 Kcal/mol for the Rh-N coordination bond.

The interaction energy of the systems, in which there are Rh(II)-coordinated NH_3 molecules and H-bonded NH_3 molecules, was determined as follows:

(Eq. 2) E(int) = E(PW_ X NH_3) – {E(PW) + X E(NH_3)} (where X is the nº of NH_3 molecules)

Table S2. Summary of interaction energies and Rh-N distance for optimized DFT structures with different amount of ammonia molecules.

Snapshot in Figure S6	Number of ammonia	E(int) (Kcal/mol)	Rh-N bond distance (Å)
**a**	1	-31.75	2.20
**b**	2	-46.30	2.18
**c**	3	-55.58	2.17
**d**	4	-66.90	2.17

Table S2 shows that each addition of NH_3 to the $Rh_2(Ac)_4(NH_3)$ complex is stabilized by *ca.* -10 Kcal/mol, which is good agreement with the stabilizing energy generally provided by H-bonding interactions.

Overall, the final configuration for the system with 4 NH_3 molecules per Rh(II)-paddlewheel cluster showed high stabilization energy and symmetry. In the most stable configuration, one NH_3 molecule is coordinated to the axial Rh(II) site and 3 NH_3 molecules are H-bonded to the $Rh_2(Ac)_4(NH_3)$. The H-bonded NH_3 molecules are distributed at 120º between each, as shown in **Figure S7**.

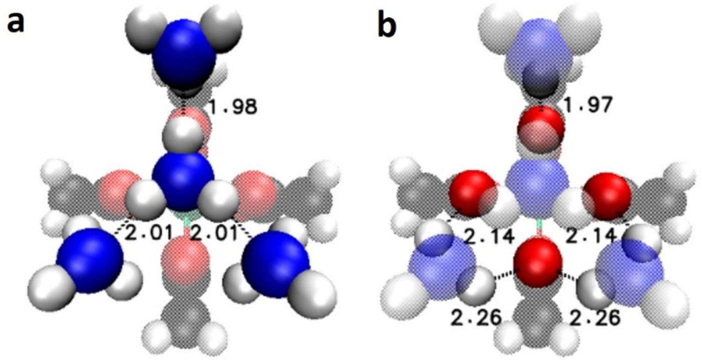

Figure S8. (a) Same structure as shown in Figure 3 of the main text but the size of the spheres was reduced to facilitate the indication of distances. Image where the NH···H H-bonding interactions are highlighted. (b) Image highlighting the COO···H H-bonding interactions.

Table S3 Summary of the distances of the H-bonds found in the optimized geometry configuration of 4 NH_3 molecules per Rh(II) site.

Interaction	Distance (Å)
$H_2N\text{-}H\cdots NH_3$	1.98
	2.01
	2.01
$H_2N\text{-}H\cdots OCO$	1.97
	2.14
	2.14
	2.26
	2.26

S5.1.2. DFT calculations of the interaction between $Rh_2(Ac)_4$, NH_3 and H_2O

In order to estimate the preference of the axial site of the Rh(II) paddlewheel for NH_3 or H_2O when the two molecules are simultaneously present, we have simulated the situation in which a Rh(II) paddlewheel is exposed simultaneously to one H_2O and one NH_3 molecule. In this simulation, H_2O and NH_3 molecules were initially located in symmetric positions from the Rh(II) paddlewheel (**Figure S10a**). Next, the geometry optimization algorithm was used to identify the minimum energy configuration, which corresponded to the situation in which NH_3 molecules occupy the axial site coordination site (**Figure S10a**). This result suggests that Rh(II)-MOPs can be efficient adsorbents for ammonia even in the presence of moisture.

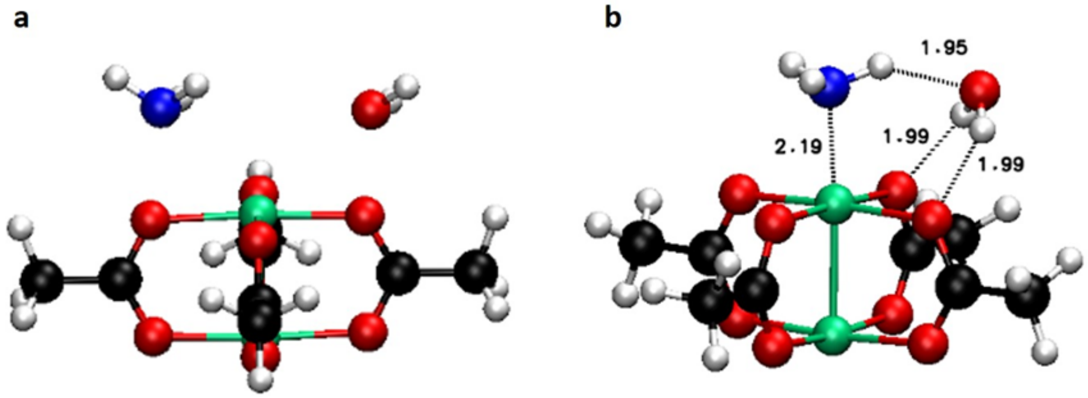

Figure S9. DFT simulation experiments used to study the preference of the Rh(II) paddlewheel for ammonia over water. (a) Initial configuration. (b) The obtained DFT optimized geometry. The DFT energy of configuration in shown in (b) is -795252.292401 kcal/mol. All molecules are shown in ball and sticks representation. Color code: carbon (black); hydrogen (white); nitrogen (blue); and oxygen (red).

S5.2. Computer simulation of the interaction between H-RhMMOP and NH_3

S5.2.1. Parametrization of the Force Field from DFT calculations

The force field employed for the molecules was based on the standard CHARMM force field,[28,29] but the parameters for the force field were modified to account for: (i) the Rh-N interaction not parametrized in the force field; and (ii) the fact that simulations take place in the gas phase (and CHARMM partial charges are derived for simulations in solvent). In general, in CHARMM force field, one includes inter-molecular nonbonding interactions given by electrostatic and Lennard-Jones 12-6 potentials and bonded interactions including harmonic bonds, angle and dihedral potentials. In our simulations, the atoms of Rh-MOP were maintained at fixed positions, and the N-H bonds in the NH_3 molecule were considered fixed so bonded interactions were not needed in our simulations. For the electrostatic and Lennard-Jones interactions, we employed the following parameters. The charges in all the atoms were determined from the DFT calculations described before using Bader charges, and they are compiled in Table S4. For the Lennard-Jones interactions, we employed the standard atom types and parameters, as defined in CHARMM. In the case of Rh (not defined in standard version of CHARMM), we employed previously proposed Lennard-Jones parameters (Rmin= 1.3575 Å and ε= -1.973 kcal/mol)[30 31] combined with standard mixing rules of CHARMM for cross interactions, except for the case of the Rh-N interaction. In that case, we parametrized the Lennard-Jones parameters to reproduce the energies and bond distances obtained in our DFT calculations. The parametrization was done in an iterative way as follows. The $Rh_2(Ac)_4$ structure with one NH_3 molecule considered in DFT calculations was now considered in NAMD in an energy minimization calculation using the above mentioned parameters for all atoms and an initial guess for the Rh-N Lennard-Jones parameters (Rmin and ε). The Rh-N bond energy was calculated from the forcefield with NAMD using a protocol analogous to that employed in DFT (see Equation 1). The obtained values of interaction energy and Rh-N bond distance were compared to DFT and the force field parameters were modified until the comparison with DFT attained a prescribed tolerance. After this optimization protocol, the obtained parameters for the Rh-N Lennard-Jones interaction were Rmin=2.20 Å and ε=-26.587 Kcal/mol. Using the final parametrization of the force field, the obtained interaction energy and bond distances were E(Rh-N)= -31.75 Kcal/mol and d(h-N)=2.20 Å, which match the DFT values.

Table S4. Bader charges calculated from DFT and partial charges employed in the molecular dynamic simulations.

Atom	Bader charges from DFT (e)	atomic charges used in MD (e)
Rh	0.58768367	0.59
O	-0.60990133	-0.61
C-type COO	0.86997117	0.87
C-type CH_3	-0.67082633	-0.671
H (in H-RhMOP)	0.242272	0.242
N (in NH_3)	-1,0676375	-1,068
H (in NH_3)	0,355879167	0,356

S5.2.2. Molecular dynamic simulations of the interaction between H-RhMOP and NH3

We run four different MD simulations to examine the interaction between H-RhMOP and NH_3. All of these simulations had one H-RhMOP in the middle of the simulation box and the same number of NH_3 molecules (457 molecules) inside the simulation box. The difference between the four simulations (named as System 1 to System 4) was the size of the simulation box, which was used to replicate the increase in NH_3 pressure that occurs during the experimental adsorption isotherm. System 1 with 400 $Å^3$; System 2 with 161 $Å^3$; System 3 with 100 $Å^3$ and System 4 with 80 $Å^3$.

The interaction of NH_3 with H-RhMOP was monitored through the radial distribution function acquired for each of the simulations (**Figure S8**). It was observed that the peak attributed to H-bonding interactions increases with the pressure of the system, whereas the Rh-N coordination remains at the same arbitrary intensity through the different simulations. From this data, it can be concluded that high NH_3 pressure favors the H-bonding interactions whereas the Rh-N coordination occurs even at low NH_3 pressure.

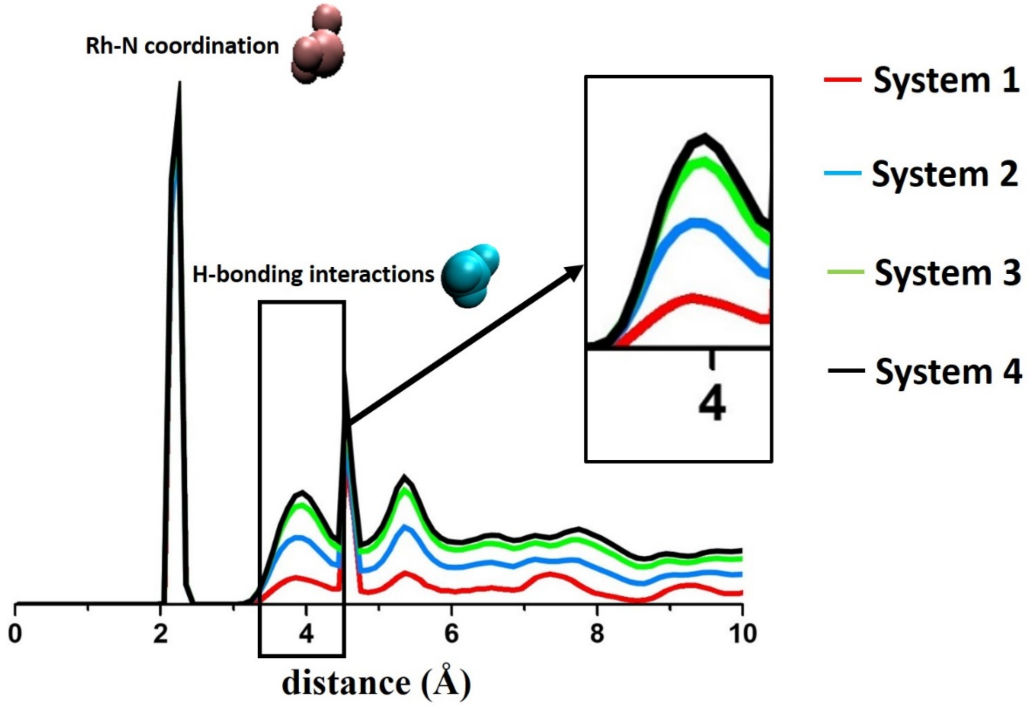

Figure S10. Radial distribution function of NH_3 and H-RhMOP (Rh atom with N atom) for each of the four different systems studied.

The number of adsorbed ammonia molecules were calculated for each system (**Table S5**), by counting the number of NH_3 molecules contributing to the first peak in the radial distribution function (i.e. to consider a NH_3 molecule as an adsorbed molecule, the Rh-N distance has to be smaller than 2.5 Å). A similar criterion was applied to identify H-bonded NH_3 molecules. In that case, the Rh-N distance is 4.5 Å.

Table S5. Number of NH_3 molecules adsorbed in H-RhMOP per type of interaction for each of the simulated systems. In all cases the total number of NH_3 molecules in the simulation is 457.

	**Nº of coordinated NH_3**	**Nº of H-bonded NH_3**
System 1	24	14
System 2	24	50
System 3	24	55
System 4	24	71

The numerical analysis of the interacting NH_3 molecules per H-RhMOP in each system simulated seems to indicate that System 3 is the one that is closest to the most stable configuration with *ca.* 4 NH_3 molecules per Rh(II) site. System 4 presents a slightly higher amount of NH_3 loading but at the expense of destabilizing H-bonding network of NH_3 molecules on top of each Rh(II) site as evidenced by the longer H-bonding distances found in this case (**Figure S9**).

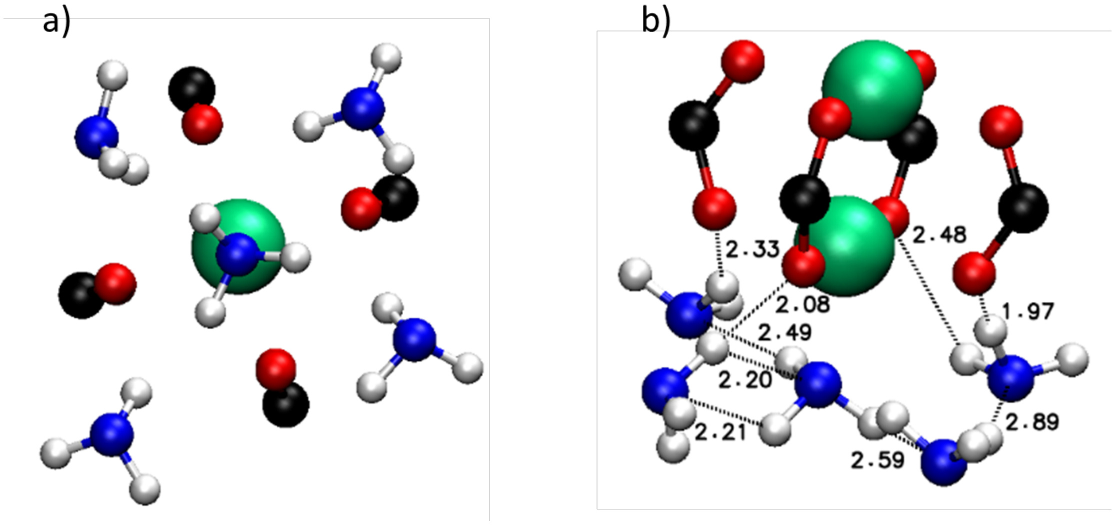

Figure S11. (a) Snapshot of the configuration of the Rh(II)-paddlewheel cluster found in System 4. (b) Alternative view of the snapshot of the configuration of the Rh(II)-paddlewheel cluster found in System 4 highlighting the intermolecular atomic distances.

S6. FTIR spectroscopy of ammonia-loaded H-RhMOP

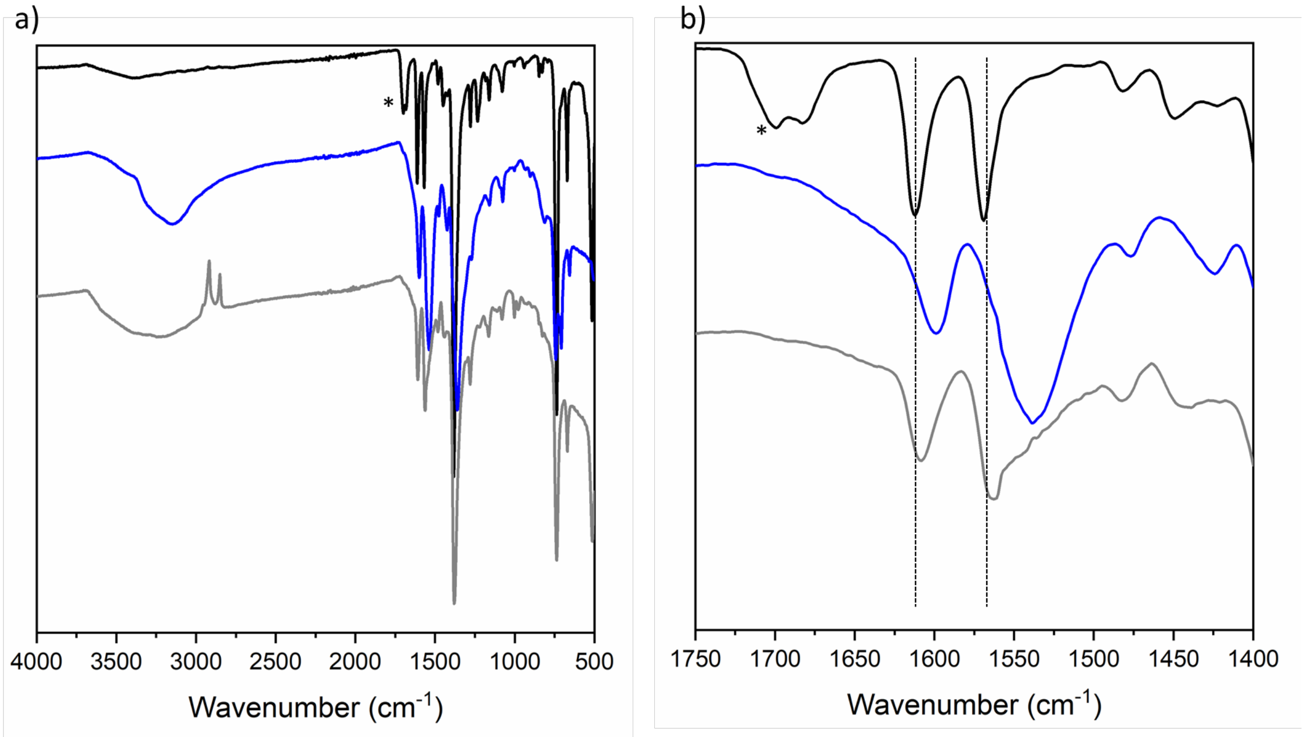

Figure S12. (a) Full FTIR spectra of as-made H-RhMOP (black), H-RhMOP after NH_3 adsorption-desorption isotherm (blue), and H-RhMOP after the regeneration process (grey). (b) Magnified area of the spectrum showing the peaks ascribed to C=O stretching bands.

S7. NH_3 uptake in $Rh_2(Ac)_4$

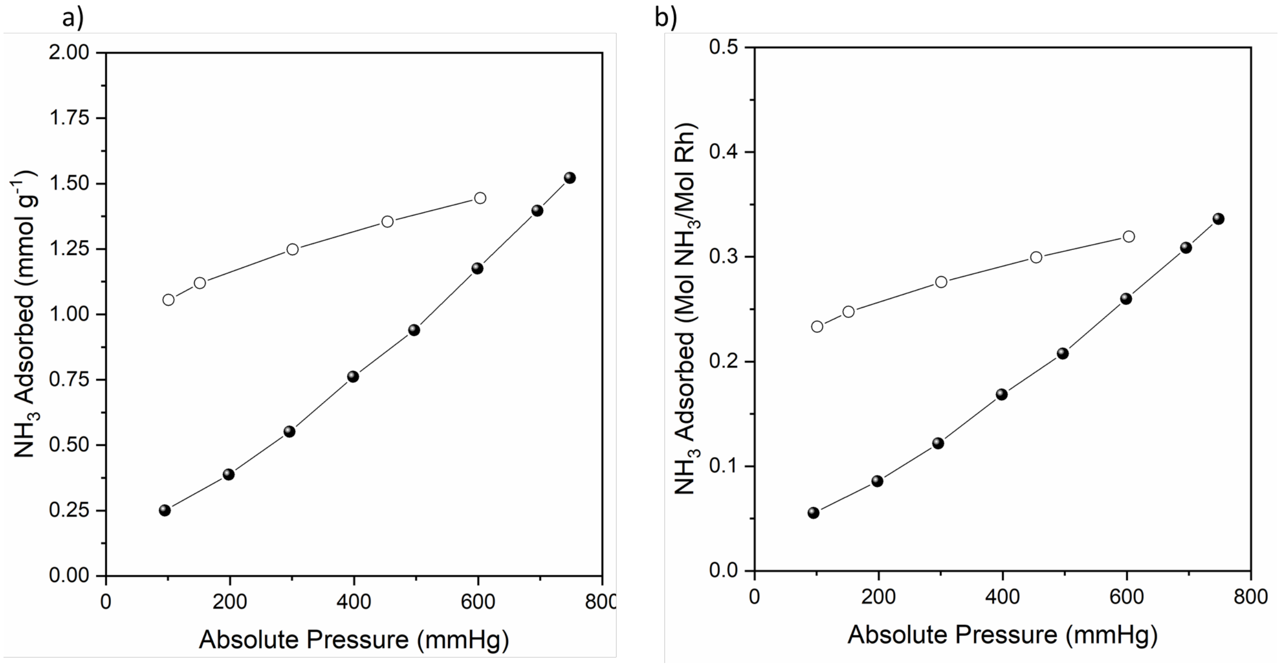

Figure S13. Ammonia-adsorption (solid dots) and -desorption (outlined dots) at 298 K of $Rh_2(Ac)_4$. (b) Shows the same ammonia-adsorption and –desorption isotherm than (a) but with the ammonia uptake values normalized per mol of Rh(II) in $Rh_2(Ac)_4$. For this calculation, the molecular weight of $Rh_2(Ac)_4$ of 442 g/mol has been considered.

S8. Digital photographs showing the regeneration of H-RhMOP

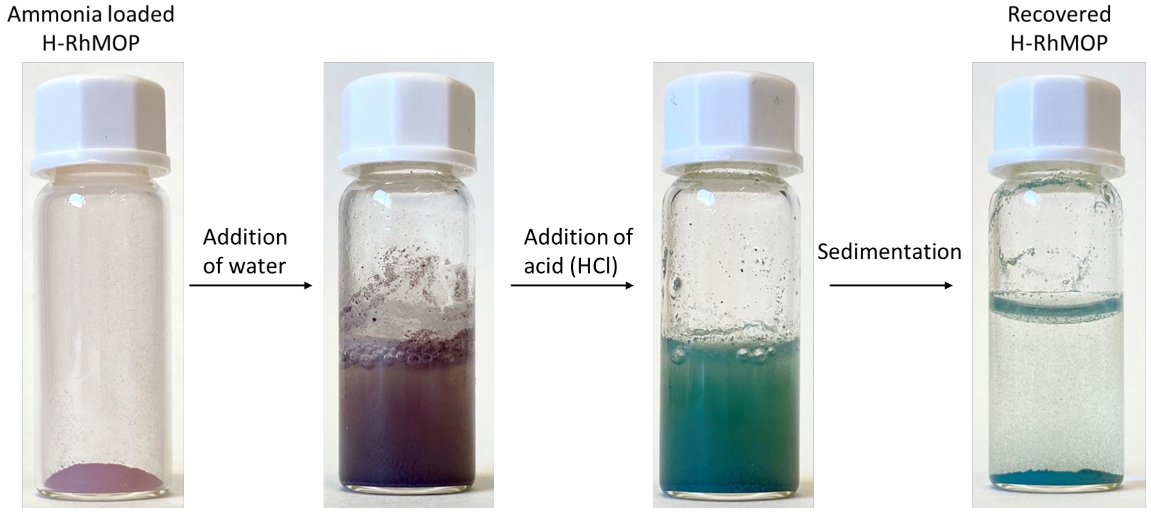

Figure S14. Digital photographs of the H-RhMOP recovery process.

S9. NH_3 uptake in OH-RhMOP and C_{12}-RhMOP

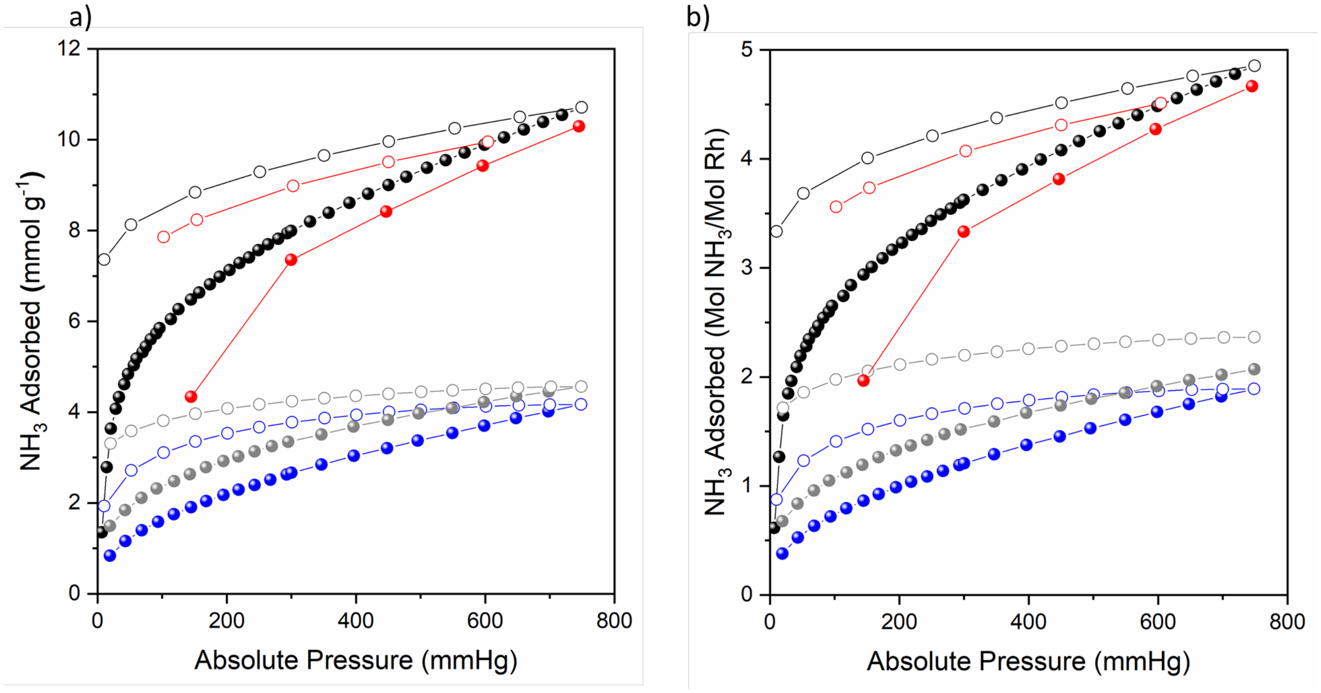

Figure S15. (a) Ammonia-adsorption (solid dots) and -desorption (outlined dots) at 298 K of pristine activated C_{12}-RhMOP (black); C_{12}-RhMOP after the first NH_3-adsorption isotherm activated under vacuum (blue); C_{12}-RhMOP after the first NH_3-adsorption isotherm activated under vacuum and heat (130º C); and regenerated C_{12}-RhMOP (red). (b) Identical ammonia-adsorption and –desorption isotherms illustrated with the ammonia uptake values normalized per mol of Rh(II) in C_{12}-RhMOP. For this calculation, the molecular weight of C_{12}-RhMOP (0.821 g/mol) was considered.

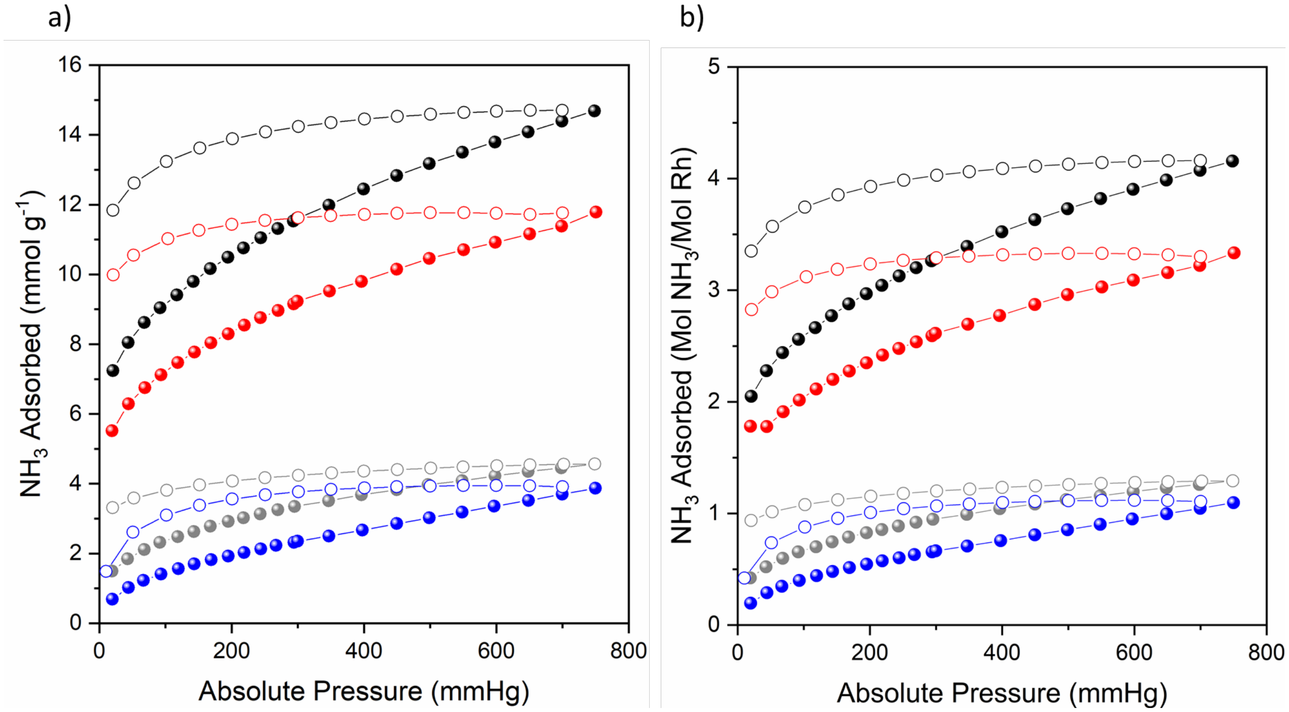

Figure S16. (a) Ammonia-adsorption (solid dots) and -desorption (outlined dots) at 298 K of pristine activated OH-RhMOP (black), of OH-RhMOP after the first NH_3-adsorption isotherm activated under vacuum (blue), of OH-RhMOP after the first NH_3-adsorption isotherm activated under vacuum and heat (130º C) and of regenerated OH-RhMOP (red). (b) Identical ammonia-adsorption and –desorption isotherms but with the ammonia uptake values normalized per mol of Rh(II) in OH-RhMOP. For this calculation, the molecular weight of OH-RhMOP (6792 g/mol) was considered.

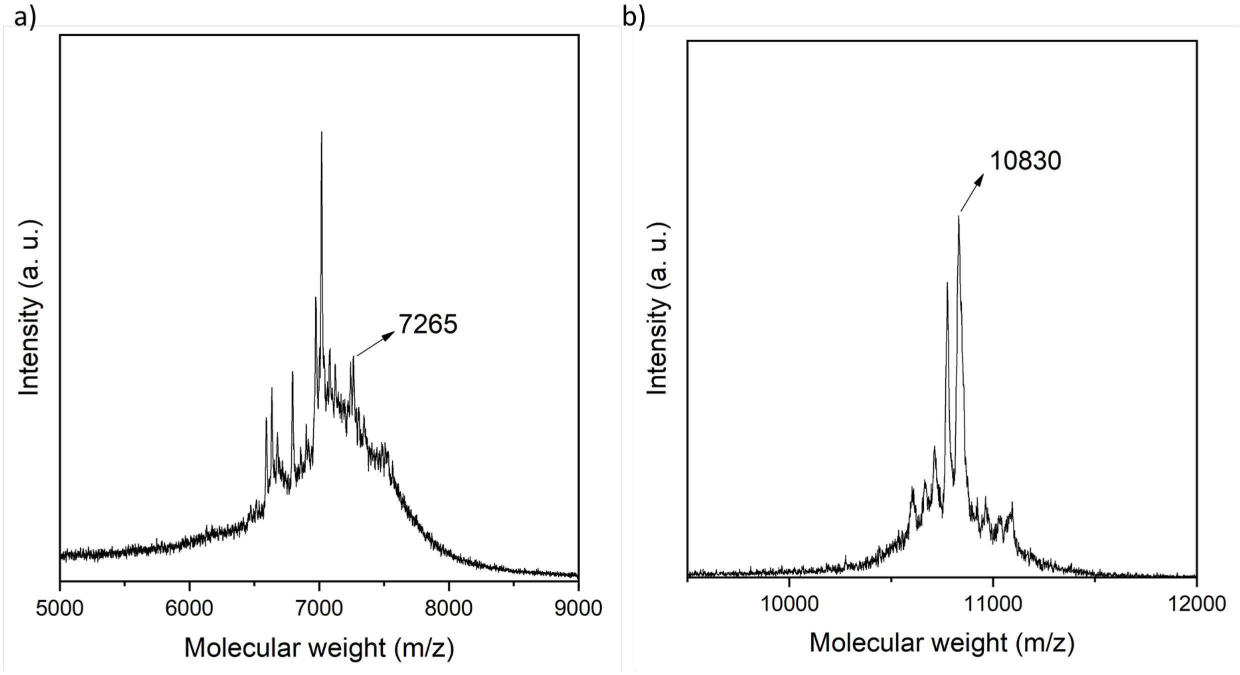

Figure S17. (a) MALDI-TOF spectrum of OH-RhMOP obtained after the ammonia sorption-desorption isotherm. OH-RhMOP was solubilized in basic water (pH = 12) to acquire the MALDI-TOF spectrum. The weight corresponding to the formula $[Rh_{24}(OBDC)_{24} + 20Na + 3H^{+}]^{-} + H_2O$ is highlighted: expected = 7269; found = 7265. (b) MALDI-TOF spectrum of C_{12}-RhMOP obtained after the ammonia sorption-desorption isotherm. The weight corresponding to the formula $[Rh_{24}(C_{12}O\text{-}BDC)_{24} + H]^{+}$ is highlighted: expected = 10822; found = 10830.

S10. Computer simulation of the interaction of functionalized Rh-MOPs and NH_3

S9.1 OH-RhMOP and NH_3

The same protocol employed for the molecular dynamic simulations of H-RhMOP and NH_3 was also employed for OH-RhMOP and NH_3 with the following results (**Figures S15 and S16**). We used the same simulation box, parameters and atoms to calculate the radial distribution function than H-RhMOP (see S5.2.3. Molecular dynamic simulations of the interaction between H-RhMOP and NH_3)

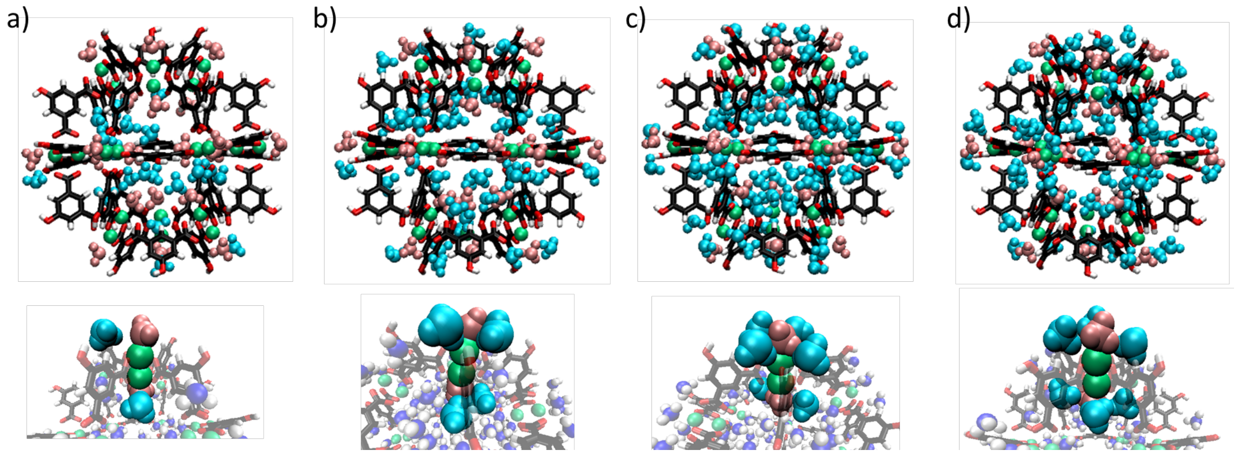

Figure S18. Top row: Screenshots of the instantaneous configurations obtained during a Molecular Dynamics simulation of a OH-RhMOP in the presence of NH_3 in system 1(a), 2(b), 3(c) and 4 (d). Bottom row: Zoomed-in images of the predominant environments of the Rh(II) paddlewheel cluster in each simulation experiment. Color code: coordinated NH_3 molecules (pink); H-bonded NH_3 molecules (cyan); and rhodium (green).

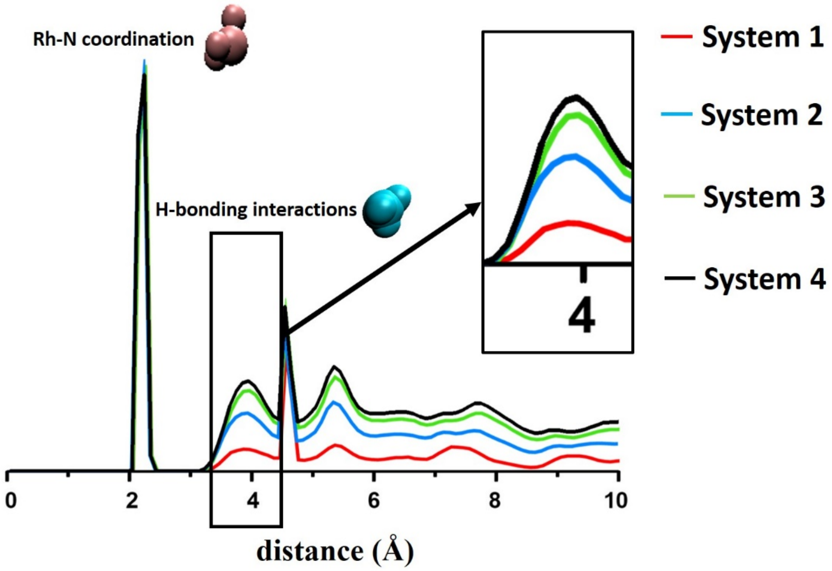

Figure S19. Radial distribution function of NH_3 and OH-RhMOP (Rh atom with N atom) for each of the four different systems studied.

A close inspection of the configurations obtained for OH-RhMOP revealed the presence of additional interactions between ammonia and OH-RhMOP beyond the ones previously described for the Rh(II) paddlewheel cluster. Specifically, we found H-bonding interactions between the hydroxyl group of the OH-RhMOP and ammonia (i.e . OH···NH_3), as depicted in **Figure S17**.

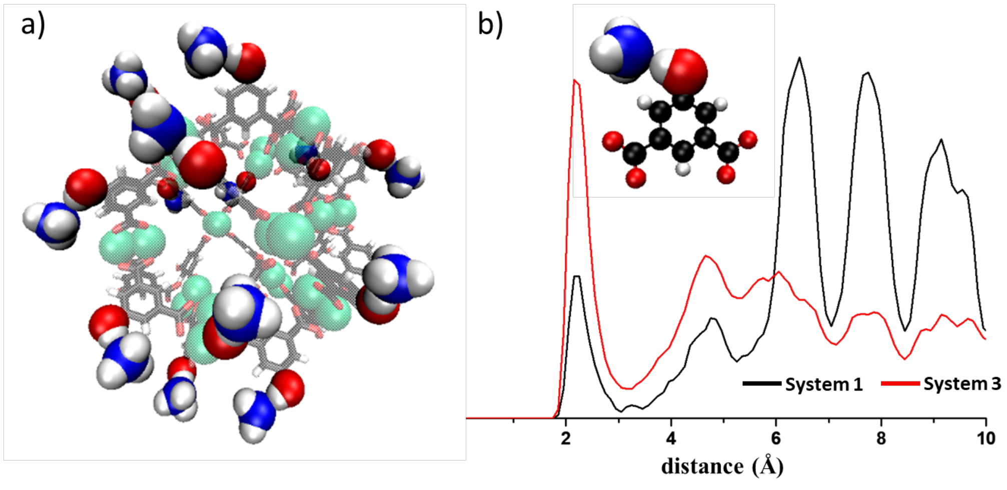

Figure 20. (a) Screen shot of an instantaneous configuration obtained in the System 4 highlighting the OH···NH_3 interactions at the surface of the OH-RhMOP. (b) The radial distribution function of N (from ammonia) and H (from -OH group of linker) for System 1 and System 3.

The number of adsorbed ammonia molecules were calculated for each system (**Table S6**), by counting the number of NH_3 molecules contributing to the first peak in the radial distribution function (i.e. to consider a NH_3 molecule as an adsorbed molecule, the Rh-N distance has to be smaller than 2.5 Å). A similar criterion was applied to identify H-bonded NH_3 molecules. In that case, the Rh-N distance is 4.5 Å. For the case of OH···NH_3 interaction, we considered a H···N distance of 2.5 Å.

Table S6. Number of NH_3 molecules adsorbed in OH-RhMOP per type of interaction for each of the simulated systems.

	**N° of coordinated NH_3**	**N° of H-bonded NH_3**	**N° of OH···NH_3**
System 1	24	19	0
System 2	24	47	4
System 3	24	60	13
System 3	24	84	17

S10.1 C_{12}-RhMOP and NH_3

Considering that we did not obtain crystallographic structure for C_{12}-RhMOP, we were not able to define the distribution of aliphatic chains in the MOP. To study the ability of oxygens from ether group to interact through H-bonding interactions, we used a simplified model to simulate the interaction of ether groups on the surface of Rh-MOPs with NH_3. Specifically, we run the simulation of System 4 on a Rh-MOP functionalized on its surface with methoxy groups in order to check if an oxygen of an ether group is able to absorb or interact with ammonia molecules (**Figure S18**). Simulation box of 80 Å^3 and 57.1 molecules/nm^3.

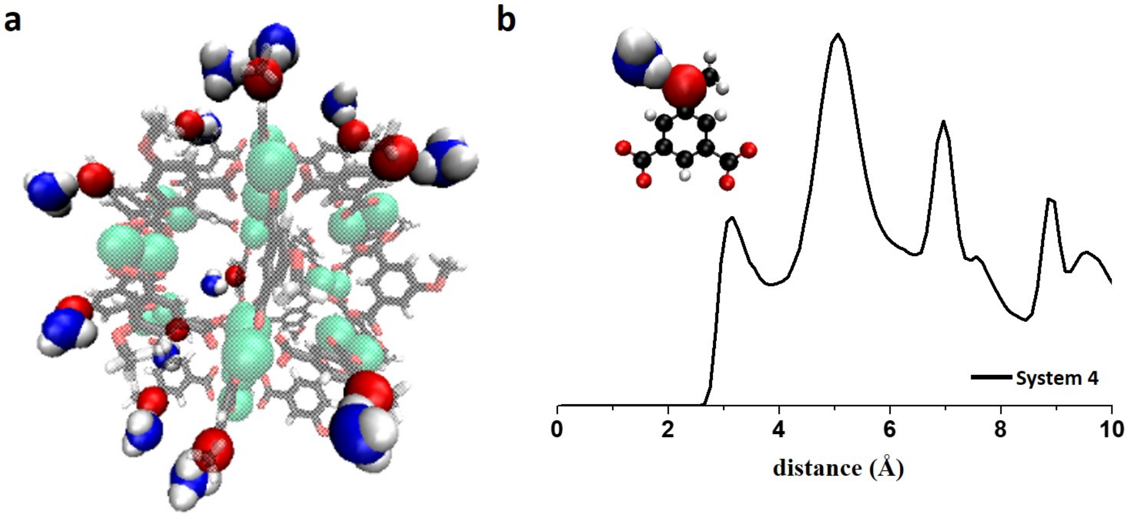

Figure 21. (a) Screen shot of an instantaneous configuration obtained in the System 4 highlighting the OMe···NH_3 interactions at the surface of the Rh-MOP functionalized with methoxy groups. (b) The radial distribution function of N (from ammonia) and O (from –OCH_3 group of linker) for System 4.

S10. References

(1) Broto-Ribas, A.; Gutiérrez, M. S.; Imaz, I.; Carné-Sánchez, A.; Gándara, F.; Juanhuix, J.; Maspoch, D. Synthesis of the Two Isomers of Heteroleptic Rh12L6L′6 Metal–Organic Polyhedra by Screening of Complementary Linkers. *Chem. Commun.* **2022**. https://doi.org/10.1039/D2CC03220A.

(2) Carné-Sánchez, A.; Albalad, J.; Grancha, T.; Imaz, I.; Juanhuix, J.; Larpent, P.; Furukawa, S.; Maspoch, D. Postsynthetic Covalent and Coordination Functionalization of Rhodium(II)-Based Metal–Organic Polyhedra. *J. Am. Chem. Soc.* **2019**, *141* (9), 4094–4102. https://doi.org/10.1021/jacs.8b13593.

(3) Hernández-López, L.; Martínez-Esaín, J.; Carné-Sánchez, A.; Grancha, T.; Faraudo, J.; Maspoch, D. Steric Hindrance in Metal Coordination Drives the Separation of Pyridine Regioisomers Using Rhodium(II)-Based Metal–Organic Polyhedra. *Angew. Chem. Int. Ed.* **2021**, *60* (20), 11406–11413. https://doi.org/10.1002/anie.202100091.

(4) Carné-Sánchez, A.; Craig, G. A.; Larpent, P.; Hirose, T.; Higuchi, M.; Kitagawa, S.; Matsuda, K.; Urayama, K.; Furukawa, S. Self-Assembly of Metal–Organic Polyhedra into Supramolecular Polymers with Intrinsic Microporosity. *Nat. Commun.* **2018**, *9* (1), 2506. https://doi.org/10.1038/s41467-018-04834-0.

(5) Frisch, M. J.; Trucks, G. W.; Schlegel, H. B.; Scuseria, G. E.; Robb, M. A.; Cheeseman, J. R.; Scalmani, G.; Barone, V.; Petersson, G. A.; Nakatsuji, H.; Li, X.; Caricato, M.; Marenich, A. V.; Bloino, J.; Janesko, B. G.; Gomperts, R.; Mennucci, B.; Hratchian, H. P.; Ortiz, J. V.; Izmaylov, A. F.; Sonnenberg, J. L.; Williams-Young, D.; Ding, F.; Lipparini, F.; Egidi, F.; Goings, J.; Peng, B.; Petrone, A.; Henderson, T.; Ranasinghe, D.; Zakrzewski, V. G.; Gao, J.; Rega, N.; Zheng, G.; Liang, W.; Hada, M.; Ehara, M.; Toyota, K.; Fukuda, R.; Hasegawa, J.; Journal of the American Chemical Society Pubs.Acs.Org/JACS Article Https://Doi.Org/10.1021/Jacs.2c06131 J. Am. Chem. Soc. 2022, 144, 15745−15753 15752 Ishida, M.; Nakajima, T.; Honda, Y.; Kitao, O.; Nakai, H.; Vreven, T.; Throssell, K.; Montgomery, J. A., Jr.; Peralta, J. E.; Ogliaro, F.; Bearpark, M. J.; Heyd, J. J.; Brothers, E. N.; Kudin, K. N.; Staroverov, V. N.; Keith, T. A.; Kobayashi, R.; Normand, J.; Raghavachari, K.; Rendell, A. P.; Burant, J. C.; Ivengar, S. S.; Tomasi, J.; Cossi, M.; Millam, J. M.; Klene, M.; Adamo, C.; Cammi, R.; Ochterski, J. W.; Martin, R. L.; Morokuma, K.; Farkas, O.; Foresman, J. B.; Fox, D. J. Gaussian 16, Revision B.01; Gaussian, Inc., Wallingford, CT, 2016.

(6) Zhao, Y.; Truhlar, D. G. The M06 Suite of Density Functionals for Main Group Thermochemistry, Thermochemical Kinetics, Noncovalent Interactions, Excited States, and Transition Elements: Two New Functionals and Systematic Testing of Four M06-Class Functionals and 12 Other Functionals. *Theor. Chem. Acc.* **2008**, *120* (1), 215–241. https://doi.org/10.1007/s00214-007-0310-x.

(7) Gallardo-Basile, F.-J. Metal Organic Frameworks: An Interplay between Experimental and Theoretical Information. Universidad de Sevilla, Https://Hdl.Handle.Net/11441/125961.

(8) Chan, W.-T.; Fournier, R. Binding of Ammonia to Small Copper and Silver Clusters. *Chem. Phys. Lett.* **1999**, *315* (3), 257–265. https://doi.org/10.1016/S0009-2614(99)01195-1.

(9) Phillips, J. C.; Braun, R.; Wang, W.; Gumbart, J.; Tajkhorshid, E.; Villa, E.; Chipot, C.; Skeel, R. D.; Kalé, L.; Schulten, K. Scalable Molecular Dynamics with NAMD. *J. Comput. Chem.* **2005**, *26* (16), 1781–1802. https://doi.org/10.1002/jcc.20289.

(10) Kim, D. W.; Kang, D. W.; Kang, M.; Choi, D. S.; Yun, H.; Kim, S. Y.; Lee, S. M.; Lee, J.-H.; Hong, C. S. High Gravimetric and Volumetric Ammonia Capacities in Robust Metal–Organic Frameworks Prepared via Double Postsynthetic Modification. *J. Am. Chem. Soc.* **2022**, *144* (22), 9672–9683. https://doi.org/10.1021/jacs.2c01117.

(11) Rieth, A. J.; Dincă, M. Controlled Gas Uptake in Metal–Organic Frameworks with Record Ammonia Sorption. *J. Am. Chem. Soc.* **2018**, *140* (9), 3461–3466. https://doi.org/10.1021/jacs.8b00313.

(12) Van Humbeck, J. F.; McDonald, T. M.; Jing, X.; Wiers, B. M.; Zhu, G.; Long, J. R. Ammonia Capture in Porous Organic Polymers Densely Functionalized with Brønsted Acid Groups. *J. Am. Chem. Soc.* **2014**, *136* (6), 2432–2440. https://doi.org/10.1021/ja4105478.

(13) Cao, R.; Chen, Z.; Chen, Y.; Idrees, K. B.; Hanna, S. L.; Wang, X.; Goetjen, T. A.; Sun, Q.; Islamoglu, T.; Farha, O. K. Benign Integration of a Zn-Azolate Metal–Organic Framework onto Textile Fiber for Ammonia Capture. *ACS Appl. Mater. Interfaces* **2020**, *12* (42), 47747–47753. https://doi.org/10.1021/acsami.0c14316.
(14) Chen, Y.; Du, Y.; Liu, P.; Yang, J.; Li, L.; Li, J. Removal of Ammonia Emissions via Reversible Structural Transformation in M(BDC) (M = Cu, Zn, Cd) Metal–Organic Frameworks. *Environ. Sci. Technol.* **2020**, *54* (6), 3636–3642. https://doi.org/10.1021/acs.est.9b06866.
(15) Han, X.; Lu, W.; Chen, Y.; da Silva, I.; Li, J.; Lin, L.; Li, W.; Sheveleva, A. M.; Godfrey, H. G. W.; Lu, Z.; Tuna, F.; McInnes, E. J. L.; Cheng, Y.; Daemen, L. L.; McPherson, L. J. M.; Teat, S. J.; Frogley, M. D.; Rudić, S.; Manuel, P.; Ramirez-Cuesta, A. J.; Yang, S.; Schröder, M. High Ammonia Adsorption in MFM-300 Materials: Dynamics and Charge Transfer in Host–Guest Binding. *J. Am. Chem. Soc.* **2021**, *143* (8), 3153–3161. https://doi.org/10.1021/jacs.0c11930.
(16) Lyu, P.; Wright, A. M.; López-Olvera, A.; Mileo, P. G. M.; Zárate, J. A.; Martínez-Ahumada, E.; Martis, V.; Williams, D. R.; Dincă, M.; Ibarra, I. A.; Maurin, G. Ammonia Capture via an Unconventional Reversible Guest-Induced Metal-Linker Bond Dynamics in a Highly Stable Metal–Organic Framework. *Chem. Mater.* **2021**, *33* (15), 6186–6192. https://doi.org/10.1021/acs.chemmater.1c01838.
(17) Moribe, S.; Chen, Z.; Alayoglu, S.; Syed, Z. H.; Islamoglu, T.; Farha, O. K. Ammonia Capture within Isoreticular Metal–Organic Frameworks with Rod Secondary Building Units. *ACS Mater. Lett.* **2019**, *1* (4), 476–480. https://doi.org/10.1021/acsmaterialslett.9b00307.
(18) Liu, J.; Lu, Z.; Chen, Z.; Rimoldi, M.; Howarth, A. J.; Chen, H.; Alayoglu, S.; Snurr, R. Q.; Farha, O. K.; Hupp, J. T. Ammonia Capture within Zirconium Metal–Organic Frameworks: Reversible and Irreversible Uptake. *ACS Appl. Mater. Interfaces* **2021**, *13* (17), 20081–20093. https://doi.org/10.1021/acsami.1c02370.
(19) Barin, G.; Peterson, G. W.; Crocellà, V.; Xu, J.; Colwell, K. A.; Nandy, A.; Reimer, J. A.; Bordiga, S.; Long, J. R. Highly Effective Ammonia Removal in a Series of Brønsted Acidic Porous Polymers: Investigation of Chemical and Structural Variations. *Chem. Sci.* **2017**, *8* (6), 4399–4409. https://doi.org/10.1039/C6SC05079D.
(20) Li, J.; Xiao, Y.; Shui, F.; Yi, M.; Zhang, Z.; Liu, X.; Zhang, L.; You, Z.; Yang, R.; Yang, S.; Li, B.; Bu, X.-H. Extremely Stable Sulfuric Acid Covalent Organic Framework for Highly Effective Ammonia Capture†. *Chin. J. Chem.* **2022**, *40* (20), 2445–2450. https://doi.org/10.1002/cjoc.202200321.
(21) Yang, Y.; Faheem, M.; Wang, L.; Meng, Q.; Sha, H.; Yang, N.; Yuan, Y.; Zhu, G. Surface Pore Engineering of Covalent Organic Frameworks for Ammonia Capture through Synergistic Multivariate and Open Metal Site Approaches. *ACS Cent. Sci.* **2018**, *4* (6), 748–754. https://doi.org/10.1021/acscentsci.8b00232.
(22) Han, Y.-S.; An, S.; Dai, J.; Hu, J.; Xu, Q.; Song, F.; Li, M.; Peng, C.; Liu, H. Defect-Engineering of Anionic Porous Aromatic Frameworks for Ammonia Capture. *ACS Appl. Polym. Mater.* **2021**, *3* (9), 4534–4542. https://doi.org/10.1021/acsapm.1c00589.
(23) Kang, D. W.; Kang, M.; Moon, M.; Kim, H.; Eom, S.; Choe, J. H.; Lee, W. R.; Hong, C. S. PDMS-Coated Hypercrosslinked Porous Organic Polymers Modified via Double Postsynthetic Acidifications for Ammonia Capture. *Chem. Sci.* **2018**, *9* (33), 6871–6877. https://doi.org/10.1039/C8SC02640H.
(24) Qajar, A.; Peer, M.; Andalibi, M. R.; Rajagopalan, R.; Foley, H. C. Enhanced Ammonia Adsorption on Functionalized Nanoporous Carbons. *Microporous Mesoporous Mater.* **2015**, *218*, 15–23. https://doi.org/10.1016/j.micromeso.2015.06.030.
(25) Helminen, J.; Helenius, J.; Paatero, E.; Turunen, I. Adsorption Equilibria of Ammonia Gas on Inorganic and Organic Sorbents at 298.15 K. *J. Chem. Eng. Data* **2001**, *46* (2), 391–399. https://doi.org/10.1021/je000273+.

(26) Lucero, J. M.; Crawford, J. M.; Wolden, C. A.; Carreon, M. A. Tunability of Ammonia Adsorption over NaP Zeolite. *Microporous Mesoporous Mater.* **2021**, *324*, 111288. https://doi.org/10.1016/j.micromeso.2021.111288.
(27) Hu, T.-T.; Liu, F.; Dou, S.; Zhong, L.-B.; Cheng, X.; Shao, Z.-D.; Zheng, Y.-M. Selective Adsorption of Trace Gaseous Ammonia from Air by a Sulfonic Acid-Modified Silica Xerogel: Preparation, Characterization and Performance. *Chem. Eng. J.* **2022**, *443*, 136357. https://doi.org/10.1016/j.cej.2022.136357.
(28) Mackerell Jr., A. D.; Feig, M.; Brooks III, C. L. Extending the Treatment of Backbone Energetics in Protein Force Fields: Limitations of Gas-Phase Quantum Mechanics in Reproducing Protein Conformational Distributions in Molecular Dynamics Simulations. *J. Comput. Chem.* **2004**, *25* (11), 1400–1415. https://doi.org/10.1002/jcc.20065.
(29) MacKerell, A. D.; Bashford, D.; Bellott, M.; Dunbrack, R. L.; Evanseck, J. D.; Field, M. J.; Fischer, S.; Gao, J.; Guo, H.; Ha, S.; Joseph-McCarthy, D.; Kuchnir, L.; Kuczera, K.; Lau, F. T. K.; Mattos, C.; Michnick, S.; Ngo, T.; Nguyen, D. T.; Prodhom, B.; Reiher, W. E.; Roux, B.; Schlenkrich, M.; Smith, J. C.; Stote, R.; Straub, J.; Watanabe, M.; Wiórkiewicz-Kuczera, J.; Yin, D.; Karplus, M. All-Atom Empirical Potential for Molecular Modeling and Dynamics Studies of Proteins. *J. Phys. Chem. B* **1998**, *102* (18), 3586–3616. https://doi.org/10.1021/jp973084f.
(30) Šebesta, F.; Sláma, V.; Melcr, J.; Futera, Z.; Burda, J. V. Estimation of Transition-Metal Empirical Parameters for Molecular Mechanical Force Fields. *J. Chem. Theory Comput.* **2016**, *12* (8), 3681–3688. https://doi.org/10.1021/acs.jctc.6b00416.
(31) Hernández-López, L.; Martínez-Esaín, J.; Carné-Sánchez, A.; Grancha, T.; Faraudo, J.; Maspoch, D. Steric Hindrance in Metal Coordination Drives the Separation of Pyridine Regioisomers Using Rhodium(II)-Based Metal–Organic Polyhedra. *Angew. Chem. Int. Ed.* **2021**, *60* (20), 11406–11413. https://doi.org/10.1002/anie.202100091.
